# Jumbo Phages: A Comparative Genomic Overview of Core Functions and Adaptions for Biological Conflicts

**DOI:** 10.3390/v13010063

**Published:** 2021-01-05

**Authors:** Lakshminarayan M. Iyer, Vivek Anantharaman, Arunkumar Krishnan, A. Maxwell Burroughs, L. Aravind

**Affiliations:** 1 National Center for Biotechnology Information, National Library of Medicine, National Institutes of Health, Bethesda, MD 20894, USA; lakshmin@mail.nih.gov (L.M.I.); ananthar@mail.nih.gov (V.A.); burrough@mail.nih.gov (A.M.B.); 2 Department of Biological Sciences, Indian Institute of Science Education and Research (IISER) Berhampur, Odisha 760010, India; akrishnan@iiserbpr.ac.in

**Keywords:** DNA viruses, anti-phage systems, nicotinamide dinucleotide, DNA polymerases, DNA polymerase III, RNA polymerase, transcription, nucleotides, virus evolution

## Abstract

Jumbo phages have attracted much attention by virtue of their extraordinary genome size and unusual aspects of biology. By performing a comparative genomics analysis of 224 jumbo phages, we suggest an objective inclusion criterion based on genome size distributions and present a synthetic overview of their manifold adaptations across major biological systems. By means of clustering and principal component analysis of the phyletic patterns of conserved genes, all known jumbo phages can be classified into three higher-order groups, which include both myoviral and siphoviral morphologies indicating multiple independent origins from smaller predecessors. Our study uncovers several under-appreciated or unreported aspects of the DNA replication, recombination, transcription and virion maturation systems. Leveraging sensitive sequence analysis methods, we identify novel protein-modifying enzymes that might help hijack the host-machinery. Focusing on host–virus conflicts, we detect strategies used to counter different wings of the bacterial immune system, such as cyclic nucleotide- and NAD^+^-dependent effector-activation, and prevention of superinfection during pseudolysogeny. We reconstruct the RNA-repair systems of jumbo phages that counter the consequences of RNA-targeting host effectors. These findings also suggest that several jumbo phage proteins provide a snapshot of the systems found in ancient replicons preceding the last universal ancestor of cellular life.

## 1. Introduction

Viral genomes span a wide size-continuum, which at the lower end includes some of the smallest natural replicons and at the higher end exceeds that of several cellular genomes. In eukaryotes, most giant viruses are unified within the diverse clade of nucleocytoplasmic large DNA viruses (NCLDVs), which includes the poxviruses, iridoviruses and asfarviruses which infect animals and several large viruses that infect microbial eukaryotes [1,2,3]. Large prokaryotic viruses first came to light with the discovery of the *Bacillus megatherium* phage G over 50 years ago, which measures more than 600 nanometers in length from head to tail, with a head diameter of just under 200 nm [4,5]. It also became clear early on that this virus is not just exemplary in virion size but also in terms of its genome [6]. This was followed by the discovery of several giant phages infecting a diverse array of bacteria. In the past two decades, these giant phages entered the “genomic era” as their genomes were completely sequenced, revealing numerous remarkable aspects of their biology [7,8,9].

Recently, the generic term jumbo phage has been applied to these giant phages, with a genome-size cutoff of 200 kb applied to include them in this category [8]. An even larger type within them, with a lower genome size cutoff of 500 kb has been termed megaphage [10]. The availability of genomic data for over 200 jumbo phages has sparked a renewed investigation of these viruses [7]. Consequently, there has been a wealth of recent studies that structurally, biochemically and ecologically characterize these viruses. One offshoot of this work has been the realization that the jumbo phages are not an evolutionarily unitary group. The tailed bacteriophages (*Caudovirales*) belong to three major structural groups: (1) The *Podoviridae* with short tails (e.g., coliphage N4); (2) the *Siphoviridae* with long non-contractile tails (e.g., coliphage lambda); (3) *Myoviridae* with long contractile tails (e.g., coliphage T4). Jumbo phages feature both *Myoviridae* and *Siphoviridae* in their ranks suggesting the independent emergence of large genome sizes in different phage groups [7]. Within these groups, the jumbo phages show a range of head morphologies which include regular icosahedra, icosahedra with elongated central triangles and icosadeltahedra. In some cases, like *Tenacibaculum maritimum* phages PTm1 and PTm5, the heads are further decorated with an assembly of fibers that project out from the top [11]. Some also display interesting variations in the tail morphology such as the presence of a mop-like array of flexible tail fibers in the *Sphingomonas* phage PAU [12] and long whiskers (*Pectobacterium* phage CBB) or hair-like fibers (coliphage 121Q and *Agrobacterium* phage Atu_ph07) [13,14] projecting from both the head and the tail-sheath. It has been proposed, although not confirmed, that these decorations might have a specific role in enhancing host attachment, especially in the context of the dispersion of virions by aqueous flow [15].

Jumbo phages have been traditionally difficult to isolate due to their large size, which prevents both easy separation from the host via filtration and diffusion in agar to form visually detectable plaques on bacterial lawns [8]. Nevertheless, the rising interest in these viruses has resulted in the identification of jumbo phages from gammaproteobacteria, betaproteobacteria, alphaproteobacteria, zetaproteobacteria, bacteroidetes, cyanobacteria, sporulating firmicutes and actinobacteria. Gammaproteobacteria are prevalent among the easily cultivable organisms, as well as those which are medically and agriculturally relevant; hence, jumbo phages infecting gammaproteobacteria are currently overrepresented in the genomic databases. While the majority of jumbo phages have been isolated from aquatic environments, they have also been found to infect bacteria from other niches such as soil, marine sediments, animal guts and plant material. More recently, metagenomic studies have augmented traditional methods of prospecting and have helped identify a range of giant phages, including the *Prevotella* megaphages in the gut microbiome [9,10]. These observations suggest that jumbo phages are likely to be far more widely distributed and numerous than the current numbers indicate. 

Jumbo phages have also sparked the interest of researchers due to several interesting aspects of their biology that have come to light from a flurry of recent studies. These include the use of multi-subunit DNA-dependent RNA polymerases (RNAPs) that are homologous to different segments of the host enzymes [16]. These homologous segments include two subunits displaying double-Ψbeta-barrel (DPBB) catalytic domains as well as separate subunits encompassing the transcript-exit clamp module and other parts of the RNAP. Several jumbo phages have two different versions of these multisubunit enzymes that are respectively packaged into the virion for early gene transcription or specialize in middle/late gene transcription [17,18]. An overlapping group of jumbo phages has been characterized as encoding a tubulin homolog, which helps form a nucleus-like compartment in the host that has been shown to protect the phage against host immune mechanisms such as the restriction-modification (R-M) and CRISPR/Cas systems by walling-off the phage replication and transcription apparatus [19,20]. Some jumbo phages have also been shown to possess their own biosynthetic capacity for NAD^+^ which is required as a substrate for phage DNA-replication and regulatory enzymes [21]. Further, some cyanophages encode their own capsular lipopolysaccharide (LPS) biosynthesis genes, which have been proposed to ward off superinfection by competing phages during periods of phage dormancy within their hosts (pseudolysogeny) [22]. Genomes of several jumbo phages also specify several diverse mechanisms that help them in their biological conflicts with their hosts such as: (1) methyltransferases that modify their DNA to evade restriction attacks on their genome. (2) Incorporation of uracil in the genome in place of thymine. (3) Arrays of tRNAs that help overcome host defense mechanisms such as those utilizing the endoRNases that restrict translation by cleaving tRNAs [7,8].

These and other findings have been surveyed in recent reviews on jumbo phages. However, there are several outstanding questions of interest that can be addressed via comparative genomic analysis and sensitive sequence and structure analysis of the phage-encoded proteins. These include the identification of various systems that deter or compensate for host immune mechanisms. More specifically, these include the complement of phage proteins that carry out RNA repair in the face of immune attacks on the translation apparatus and the phage enzymes that might modify DNA and RNA beyond the previously described DNA methylases. The jumbo phage enzymes akin to the coliphage T4-encoded ADP ribosyltransferases that help in modifying and hijacking host systems have also not been studied closely [23]. Further, it has recently become apparent that bacteria deploy several effector systems activated by nucleotides and NAD^+^ derivatives to target viruses [24]. The complement of phage countermeasures against such systems remains poorly explored. In the current article, we carry out a systematic comparative genomic survey of jumbo phages to address these questions. Consequently, we identify diverse phage systems that are likely to form interfaces of the conflict with their hosts. We also use this information to throw light on less-appreciated aspects of various previously studied systems such as the DNA replication and repair enzymes, RNA polymerases, and different transcriptional strategies used by the jumbo phages.

Moreover, this investigation helps understand the independent, parallel growth of genome size in several viral lineages. To this end, we have tried to objectively define the genome size criteria for giant phages rather than using an arbitrary cutoff of 200 kb. The independent evolution of jumbo phages infecting diverse bacteria and also the presence of certain parallels to their eukaryotic counterparts, the NCLDVs, suggests that large genome sizes represent a potential strategy that has repeatedly emerged under natural selection independently of the type of host. This phenomenon might have general implications for the dramatic variations in genome size seen among both viruses and cellular organisms.

## 2. Materials and Methods

### 2.1. Sequence Analysis

Sequence profile searches were performed using the PSI-BLAST (RRID: SCR_001010) [25] and JACKHMMER programs (RRID: SCR_005305) [26] with the profile being built at each iteration. Clustering for both classification and purging of nearly identical sequences was performed with the BLASTCLUST program (version: 2.2.26, NCBI, USA, ftp://ftp.ncbi.nih.gov/blast/documents/blastclust.html) (RRID: SCR_016641 with the length of the pairwise alignment (L) and measure of similarity, i.e., bit-score (S) adjusted depending on the degree of clustering required. 

HMM searches were run using either HMMsearch initiated with an HMM built from an alignment or iteratively using JACKHMMER from single seeds [26]. Sequence searches were run against either the non-redundant (nr) protein database or custom databases of completely sequenced 224 jumbo phages or 10,176 *Caudovirales* available in Genbank as of 10 October 2020. Names of the phages were as provided in the taxonomy division of Entrez (https://www.ncbi.nlm.nih.gov/Taxonomy/Browser/wwwtax.cgi?id=28883). To obtain the proteome of the *Prevotella* megaphage Lak-B8, the nucleotide sequence deposited in Genbank was translated using Prodigal [27] using the translation table codes 11 and 15 as mentioned in the genome publication [10]. Profile-profile searches were conducted using HHpred (RRID: SCR_010276) and run against (1) HMMs derived from PDB; (2) Pfam models; (3) A custom database of alignments of diverse domains curated by the Aravind group [28]. All novel alignments that were added to this database are provided in the (Supplementary Materials S2). Multiple sequence alignments were built using Kalign (162) (RRID: SCR_011810) and Muscle with manual adjustments based on profile-profile and structural alignments [29,30]. Secondary structures were predicted using the JPred program (RRID: SCR_016504) [31].

### 2.2. Structure Analysis

Structure similarity searches were performed using the DaliLite program (RRID: SCR_003047) run against the PDB database clustered at 75% sequence similarity [32]. Structure similarity trees were constructed based on Z-scores obtained from an all-vs-all search of the compared structures using average linkage clustering. Structural visualization and manipulations were performed using the PyMol (http://www.pymol.org) (RRID:SCR_000305) and MOL* programs (http://molstar.org) [33]. The structural figure panels were rendered by extracting the state PDB IDs and presenting them in the cartoon or molecular surface views with the MOL* program and colored to emphasize relevant features.

### 2.3. Comparative Genomics

Taxonomic lineages were obtained from the NCBI Taxonomy database (see Section 2.1). Contextual information from prokaryotic gene neighborhoods was retrieved using a Perl script to extract upstream and downstream genes of the query gene from the GenBank genome file. Their products were then clustered with BLASTCLUST to identify conserved gene-neighborhoods based on conservation between different taxa. Several additional filters were then applied to recognize valid neighborhoods for further analysis: (1) nucleotide distance constraints (generally 50 nucleotides); (2) conservation of gene directionality within the neighborhood; (3) presence in more than one phylum. Phylogenetic trees were constructed using an approximate maximum-likelihood method implemented in the FastTree 2.1 (RRID: SCR_015501) program under default parameters [34].

The phyletic patterns were used to generate two sets of vectors, namely the distribution by phage for a given protein and the complement of proteins for a given phage. These were used to compute the inter-protein or inter-phage Canberra distance [35], which is best suited for vectors with integer data in the form of presences and absences. The Canberra distance between two vectors $\vec{p}$ and $\vec{q}$ is defined as:

$$d(\vec{p},\vec{q})=\frac{\left|p_{i}-q_{i}\right|}{\left|p_{i}\right|+|q_{i}|}$$

These distances were used to cluster the protein families and phages through agglomerative hierarchical clustering using Ward’s method [36]. Ward’s method takes the distance between two clusters A and B, as the amount by which the sum of squares from the center of the cluster will increase when they are merged. Ward’s method then tries to keep this growth as small as possible. It tends to merge smaller clusters that are at the same distance from each other as larger ones, a behavior useful in lumping “stragglers” in terms of both phages and proteins with correlated phyletic patterns. The same vectors for phages were also used to perform principal component analysis to detect spatial clustering by dimensionality reduction. The variables were scaled to have unit variance for this analysis. Data processing (knitr and dplyr libraries), network analysis (igraph library), and visualization were performed using the R language.

A complete annotated list of all the jumbo phage protein clusters with domain architectures as classified in this study is available as Supplementary Materials (Supplementary S5). Any protein family referred to in the text may be found by searching the Supplementary table with the representative accession provided in the text.

## 3. Results and Discussion

### 3.1. The Basic Features of Genome Size and Protein Length Distributions of Giant Phages

**Figure 1:**
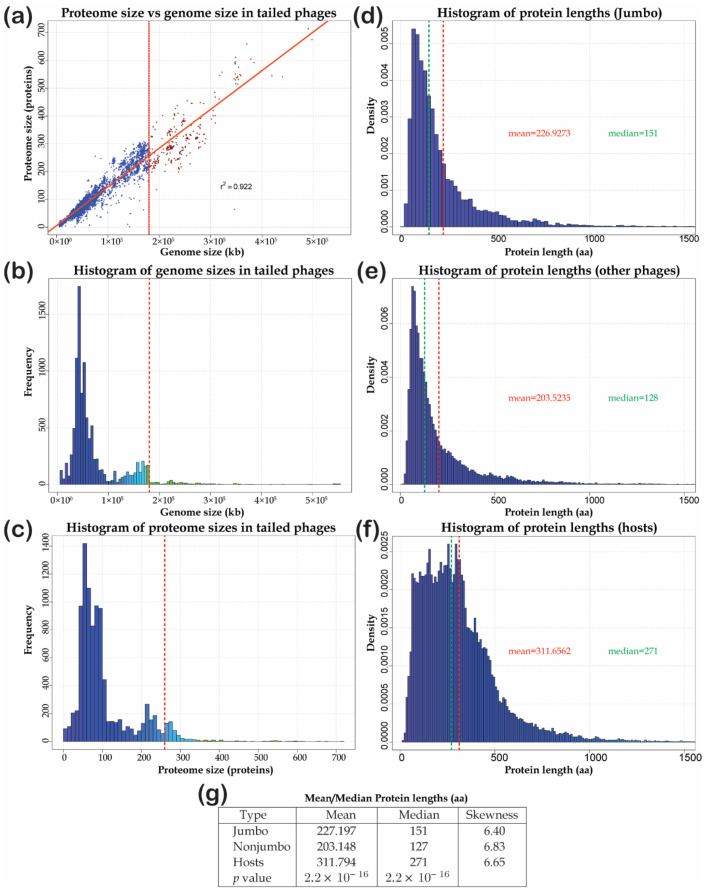
Bulk genome and proteome features of *Caudovirales*, jumbo phages and their hosts. Various genome and protein features of *Caudovirales* are illustrated including (**a**) protein density; (**b**) genome and (**c**) proteome size distributions. In (**a**) points are colored blue if the genome size <180 kb and red if >180 kb. Protein length distributions are illustrated for (**d**) jumbo phages (**e**) non-jumbo phages and (**f**) those of their hosts. The length distribution statistics are summarized in (**g**). The red vertical line in (**a**–**c**) shows the objective cutoff used to define the jumbo category in phages. Panels (**b**) and (**c**) use a color palette ranging from dark blue to green (R language: topo.colors function) to color each bar in the histogram.

To get a handle on the distribution of genome (proteome) sizes, we assembled a database of 10,176 *Caudovirales* with complete genomes and plotted the sizes of their predicted proteome against their genome size (Figure 1a). The proteome size ranging from 1 to 714 proteins is strongly positively and linearly correlated with genome size (r^2^ = 0.922, *p* = 2 × 10^−16^) ranging from 5 kb to 551 kb. This suggests that despite the wide size range, proteins are encoded at similar densities across phage genomes. Consistent with this, a plot of the density of protein-coding genes on phage genomes shows a tight approximately normal distribution with a mean of 1.5 genes (standard deviation 0.25) per kb of the genome (Figure 1a).

This suggested that the histogram of the distribution of phage genome or proteome sizes would be useful to determine any objective size categories that might exist. These plots reveal a trimodal distribution with distinct valleys between the peaks dividing the phages into three size categories (Figure 1b,c). The first and most abundant category consists of the small phages with genome size less than 100 kb (modal value ~50 kb) and coding for less than 150 proteins. This is followed by the category of next abundance comprised of medium-sized phages with genome sizes less than 180 kb and typically coding for less than 250 proteins. The final category is relatively sparse with genomes larger than 180 kb with a long right tail but a current modal value of around 230 kb. While sampling bias could account for some of the differences, the modality in the distribution points to the existence of a genuine giant-phage category with genome sizes greater than 180 kb (Figure 1a,b). As compared to the cut-off of greater than 200 kb genomes employed in studies on jumbo phages, our cutoff includes 45 additional phages in the jumbo phage category with an average proteome size of 259 proteins. A recent study of phages from diverse environmental samples found diverse giant phages (with genomes greater than 200 kb) to be lodged in larger clades with genomes of at least 120 kb in length [9]. This illustrates the tendency of the jumbo phages to belong within larger clades with at least medium-sized genomes. Therefore, the above-proposed cutoff based on the size distribution helps more objectively define the genomic size category that includes the jumbo phages and is treated as the focus of our analysis. At the lower end of this category are coliphages like RB43, while the higher end features phages such as the *Prevotella* megaphage Lak-B8, *Bacillus* virus G, *Agrobacterium* hairy phage Atu_ph07 and phage SCTP-2 which infects the hyperhalophilic gammaproteobacterium *Salicola*. 

A study of the distribution of the lengths of proteins in amino acids also suggests that this category can be discriminated on an average from the other phages (Figure 1d–f). The lengths of all proteins encoded by small/medium-sized phages, jumbo phages and their host genomes show similar unimodal distributions with a comparable prominent right skew (skewness = 6.4–6.8; Figure 1). However, there are differences in the median protein length with the viruses having significantly lower median protein lengths than their cellular counterparts (Kruskal–Wallis test *p* = 2 × 10^−16^) (Figure 1d,e). Within the phages, the jumbo phages have significantly longer median lengths of proteins (127 vs. 151, *p* = 3.2 × 10^−13^) than the rest. Therefore, among these DNA viruses, there is a trend for longer proteins going along with their larger genomes. Thus, while the phages with genome sizes of 180 kb or more morphologically conform to the same categories as the small/medium-sized phages, they can be defined as a distinct group encompassing the jumbo phages based on the above criteria.

### 3.2. Conserved Jumbo Phage Proteins Define Distinct Groups with Multiple Independent Origins

**Figure 2:**
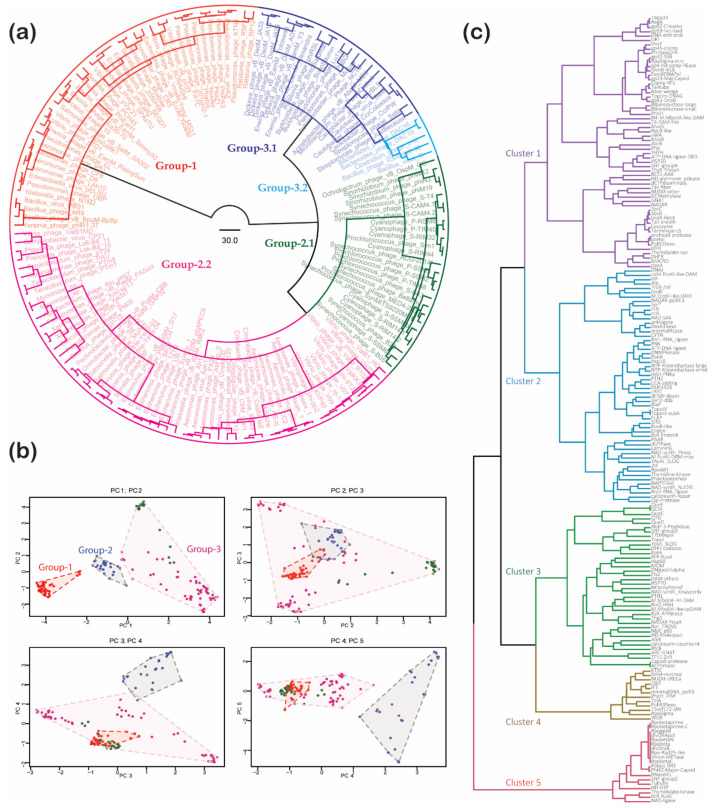
Phylogenetic analysis of jumbo phages and their proteins. Phyletic patterns of 113 protein families were used to (**a**) compute the Canberra distance for Ward’s clustering to construct a dendrogram of phages, and (**b**) for Principle Component Analysis for 154 jumbo phages with unique phyletic patterns. The convex hulls bounding the groups are shown on the Principal Component Analysis (PCA) plots. Members groups 1, 2.1, 2.2, 3.1 and 3.3 are colored distinctly. Organisms are colored by groups as in (**a**). The Canberra distance and Ward’s clustering were also used to compute the (**c**) protein dendrogram for 181 protein families which illustrates proteins sharing similar phyletic patterns.

Unlike cellular genomes, there are few proteins universally conserved across jumbo phages. The terminase large subunit, the DNA-packaging protein characteristic of *Caudovirales*, comprised of a N-terminal DNA-pumping P-loop ATPase domain and a C-terminal RNase H fold endonuclease domain, is universal. The chromosome-end-processing complex comprised of the ABC ATPase (ortholog of cellular SbcC) and its nuclease partner SbcD belonging to the calcineurin-like phosphoesterase superfamily are practically universally conserved. Further, proteins with a lysozyme fold that assist phages in degrading the sugar linkages of cell walls are also nearly universal. Beyond these, all other conserved proteins show more patchy phyletic patterns across jumbo phages. In part, this arises from extreme sequence divergence, especially in the case of certain virion structural proteins. A recent study has defined phage clades using the universal terminase subunit and acknowledged that the different conserved proteins yield different tree topologies [9]. We found that the terminase large subunits from different phages can evolve at rather distinct rates; for instance, the terminase subunit of the *Prevotella* megaphage Lak-B8 is evolving at a distinctly higher rate than those from some of the other jumbo phages. In addition to the evidence for different evolutionary rates of conserved proteins, which is typical of viruses, there are also genetic exchanges between distantly related clades. Hence, conventional phylogenetic trees based on single or concatenated protein alignments are not effective in revealing the higher-order evolutionary relationships and recombinational events shaping viral genomes. Therefore, we instead used clustering and principal component methods based on phyletic pattern vectors of conserved proteins from jumbo phages to obtain a picture of their relationships (Figure 2).

To this end, we performed single-linkage clustering of all the proteins from 224 representative jumbo phages based on BLAST pairwise-alignment bit scores to identify various protein families found in them. These families were then searched using sequence profiles and hidden Markov models with the RPS-BLAST [25] and HMMSCAN (HMM3 package) [26] programs to identify conserved domains. In cases where such domains could not be identified by these methods, we carried out iterative sequence-profile, hidden Markov model searches and profile-profile searches in an attempt to identify any further conserved domains. As a result, we obtained a comprehensive collection of domain architectures for the protein families found in jumbo phages.

Using the phyletic patterns of 113 such families with wide representation in jumbo phages, we computed a distance matrix using the Canberra distance and derived a dendrogram of phages (Figure 2a) using the Ward clustering algorithm (see Methods). The same phyletic pattern matrix was also used for principal component analysis (Figure 2b) to identify potential groupings of phages by plotting pairs of the first five principal components that explain 63% of the variance.

The above procedures revealed that at the highest level the jumbo phages can be divided into three broad groups (Figure 2a). Group 1 includes the classic jumbo phages prototyped by the *Pseudomonas aeruginosa* phage PhiKZ. This group is typified by the presence of the multi-subunit “double-barrel” RNAP, an unusual DNA polymerase from a unique and divergent clade of family B DNA polymerases, a divergent version of the DnaB-helicase (see below) and a PhiKZ-type major capsid protein. This group broadly follows the PhiKZ functional model. Group 2 is characterized by a classic family B DNA polymerase, a classic OB-fold single-strand binding protein, a phage T4 UvsW/poxviral A18 type DNA helicase, the absence of an RNAP but the presence of phage-encoded sigma factors and the distinct gp23-type major capsid protein. Group 2 further splits up into two subgroups: 2.1 comprises of phages infecting both cyanobacteria (e.g., *Synechococcus* phage S-Cam4.1) and alphaproteobacteria (e.g., *Ochrobactrum* phage vB_OspM_OC); 2.2 includes phages mostly infecting gammaproteobacteria, the alphaproteobacteria (e.g., Atu_ph07) and bacteroidetes (e.g., the *Prevotella* megaphage LAK-B8 and *Tenacibaculum* phage PTM1). Group 3 is further split into two, of which 3.1 is defined primarily by the presence of a coliphage T7-type DNA polymerase, whereas group 3.2 (typified by the *Bacillus megatherium* phage G and *Clostridium* phage c-st) is defined by the presence of a DNA polymerase III type enzyme similar to the primary replicative enzyme of bacteria. Whereas groups 1 and 2 are entirely made up of *Myoviridae*, group 3 includes both *Myoviridae* and *Siphoviridae*. The *Myoviridae* across all groups are unified by the presence of a tail-sheath protein which forms part of the contractile tail typical of these viruses (Figure 2a). *Aeromonas* phage AP1 shows hybrid features of both groups 1 and 2.2; however, the group 1 proteins in this phage are nearly identical to other *bona fide Aeromonas* group 1 phages, raising the question of contamination of the genome assembly. 

The characteristic proteins of each group of jumbo phages are also found in certain medium-sized phages; for instance, the group 2 phages are the jumbo phages related to the coliphage T4. Thus, group 2 phages can be seen as arising via genome expansion from coliphage T4-like predecessors. This is consistent with a recent study that found jumbo phages from environmental samples to be lodged in clades with other medium-sized phages [9]. Together, these observations indicate that there have been at least 6–7 distinct origins of jumbo phages from smaller precursors. Moreover, the presence of both *Siphoviridae* and *Myoviridae* in group 3 suggests that there has been a recombinational exchange of core machinery between viruses with distinct virion function (contractile vs. flexible tails) and morphology. This recombinational exchange is also borne out by examples such as the ATP- and NAD^+^-dependent DNA ligases shared by mutually exclusive subsets of jumbo phages (Figure 2c).

### 3.3. Phyletic Patterns Define Correlated and Complementary Functional Systems in Jumbo Phages

Phyletic patterns have served as a tool to objectively define potential functional linkages based on the co-occurrence, shared absences and complementary patterns of proteins. Accordingly, we clustered 181 different protein families identified in jumbo phages using the Canberra distance and Ward algorithm to detect potential function connections between them (Figure 2c). As a test of its effectiveness for detecting functional linkages, we examined the clusters for certain known functional associations. For example, the individual components of the multi-subunit RNAP group together keeping with their forming a protein complex. Similarly, we found a grouping of dihydrofolate reductase (DHFR) and thymidylate synthase (TS) superfamilies (Figure 2c). It is known that several phages sustain their DNA replication by synthesizing their own thymidylate or a modified base through the transfer of a single carbon-atom fragment (a methyl or hydroxymethyl group) to uracil catalyzed by thymidylate synthase [37,38]. This single carbon moiety is borne on a tetrahydrofolate cofactor which in turn is produced by DHFR; thus, their grouping in the dendrogram recapitulates their functional association. In contrast, the classic TS is anticorrelated with ThyX which is a non-homologous enzyme with the same activity [39]. Thus, the majority of jumbo phages have either a member of the TS or ThyX superfamilies, indicating that they produce their own thymidylate or a modified pyrimidine.

The cluster analysis also pointed to some subtler functional connections. For example, tubulin is correlated in its phyletic pattern with the multi-subunit RNAP (Figure 2c). This suggests that the formation of the nucleus-like compartment went hand-in-hand with the acquisition of host-like transcription machinery that allowed independence from the host transcription apparatus, which is likely kept out by the sub-cellular compartment formed by the tubulin within which phage replication and transcription occurs. The same set of phages also frequently code for a member of the HSP60 superfamily of ATP-dependent chaperones (Figure 2c). This raises the possibility that the chaperone might aid in the (dis)assembly of the nucleus-like compartment in the course of the phage cycle. Curiously, the co-chaperone of HSP60, HSP10 is found in an entirely different set of phages from groups 2 and 3, in which it might recruit the host HSP60 as a partner for virion assembly. Both group 1 and group 2 phages use the prohead serine peptidase of the SH superfamily (Pfam S77); obvious homologs of these are not found in group 3 jumbo phages. However, we observed that group 3 jumbo phages show a complementary pattern to this prohead peptidase with either an unrelated XkdF family serine peptidase [40] or a divergent clade of SH superfamily peptidases [41] (AYD81192.1). This suggests that these might be the equivalent head-protein processing peptidases in this group (see Supplementary Materials S1.1–1.11 for a comprehensive collection of phyletic patterns).

Beyond these, we found several other examples of functional correlations and complementary patterns relating to proteins in DNA replication, transcription, DNA modifications and metabolism. We consider these below categorized by functional systems along with a more detailed discussion on the domain architectures of the proteins under consideration.

### 3.4. Major Functional Categories of Jumbo Phage Proteins

The infection cycle of jumbo phages displays the same temporal landmarks and functional modules typical of other lytic *Caudovirales* [42]. In *Myoviridae*, the first step, namely invasion, involves the contractile mechanism of the tail-sheath protein with the active injection of the DNA into the host cell via the tail-tip proteins upon engagement of the tail spike proteins with the cell-surface receptors and degradation of the peptidoglycan by lysozymes such as gp13 [43]. The flexible tails of *Siphoviridae* lack a contractile tail-sheath; here, the tape measure protein is believed to form a conduit to deliver the DNA through the host cell wall [43]. The DNA might be delivered along with the accompanying “pilot” proteins (e.g., the RNA polymerase) that help establish the virus once inside the host. The core lytic cycle is initiated by the successive transcription of early, middle and late genes mediated by different transcription factors and or RNA polymerases [42]. Among the products of the late genes, are the replication and recombination proteins on one hand and the structural and DNA-packaging proteins on the other. Together, these complete the replication of the viral DNA followed by its packaging into the head in an ATP-dependent manner and assembly of the complete virion prior to lysis of the host cell. However, there is increasing evidence that this basic lytic cycle might be paused in several jumbo phages for a “pseudolysogenic” phase depending on the environmental stress status of the host [44]. Further, right from the point of the injection of DNA to lysis of the host cell, the virus and the host are locked in a biological conflict. On the host side, this involves the deployment of a diverse array of phage restriction mechanisms, whereas on the virus side it involves an equally diverse array of enzymatic and non-enzymatic mechanisms to hijack host machinery and counter the restriction mechanism.

Rather than discuss the functional categories of jumbo phage proteins sequentially as per the above-summarized infection cycle, we discuss them in the order of their importance in distinguishing the different groups of jumbo phages and the presence of novel biochemical features with respect to the smaller phages. Consequently, we first cover the basic DNA replication paradigms (Section 3.4.1) and recombination apparatus (Section 3.4.2) followed by the transcription apparatus (Section 3.4.3). These are followed by sections on the strategies used to hijack host systems (Section 3.4.4) and virion structure and assembly (Section 3.4.5). Since the latter of these categories generally resembles smaller *Myoviridae* and *Siphoviridae*, and has been covered in depth before [43], in this subsection we restrict ourselves to previously unreported or underappreciated findings from jumbo phages. These categories are central to distinguishing the higher-order groups of jumbo phages. These are followed by five subsections devoted to the strategies used to counter host defenses (Section 3.4.6, Section 3.4.7, Section 3.4.8, Section 3.4.9 and Section 3.4.10) which present several new observations on these systems.

#### 3.4.1. Proteins that Define the Four Basic DNA Replication Paradigms in Jumbo Phages

**Figure 3:**
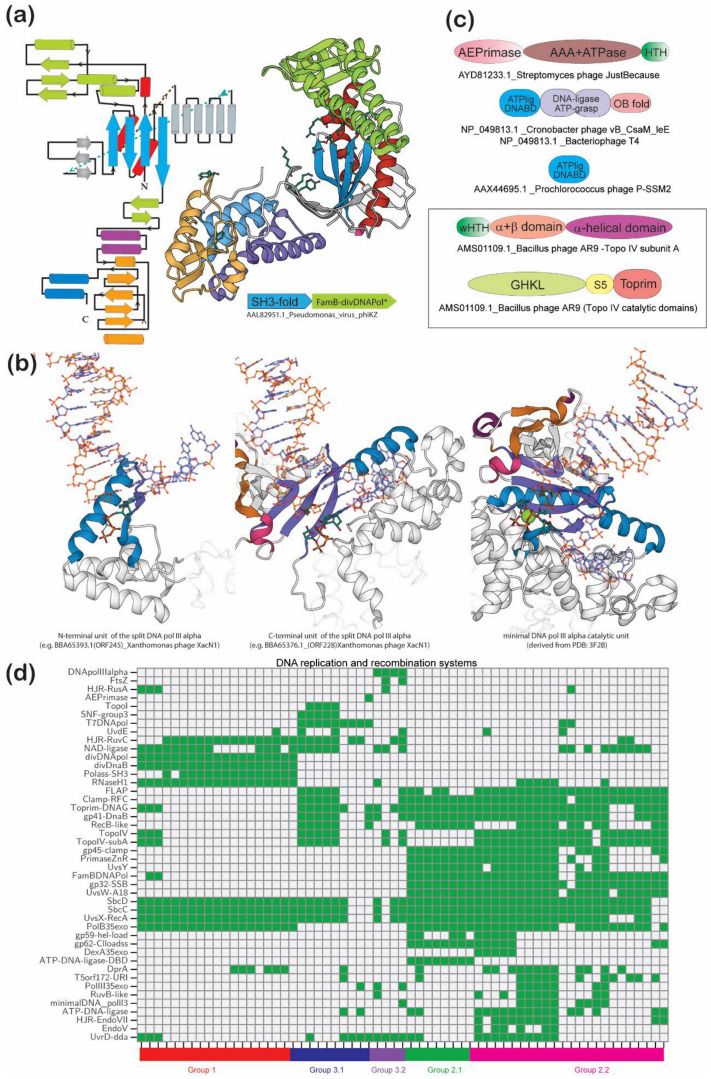
DNA replication and recombination systems of jumbo phages. (**a**) Predicted topology diagram, cartoon structure, and most common gene neighborhood of the divergent DNA polymerase of group 1. In the topology diagram, helices are shown as cylinders and strands as arrows. The cartoon structure was rendered using the MOL* program. Corresponding secondary structure elements are colored similarly in the topology diagram and cartoon. Genes are represented as boxed arrows with arrow-heads pointing to the 3′ ends of the gene. The accession number in the label corresponds to the gene marked with a *. (**b**) Predicted components of the split DNA Polymerase III catalytic α subunit. The N-terminal unit is shown on the left and the C-terminal in the middle. (**c**) Domain architectures of some interesting proteins involved in DNA replication and recombination. Proteins are denoted by their accession numbers and phage names separated by underscores. (**d**) Phyletic vector diagram of various replication and recombination proteins in a sample of jumbo phages. The Canberra distance and Ward clustering were used to determine protein and species order in the matrix. Filled green squares indicate presence of a gene. The phage name order is the same as in Figure 4 in all vector diagrams in the main figures.

One of the primary distinguishing features of the three higher-order groups of jumbo phages is their distinct DNA replication apparatus. All jumbo phages possess their own DNA polymerase, suggesting that they are self-sufficient with respect to their core replication apparatus (Figure 3). Jumbo phages possess one or more of five distinct types of DNA polymerases; three of them have a related core catalytic domain, termed the palm domain that adopts the “RNA-recognition-motif-like” (RRM) fold with four strands and two helices [45,46]. Two active site Mg^2+^ ions are chelated by charged residues from the end of the first strand and the β-hairpin formed by the 2nd and 3rd strands of the core RRM fold (Figure 3a). Strand 1 is followed by an insert region termed the finger module that plays a role in binding the template nucleic acid [46,47]. The remaining two jumbo phage DNA polymerases are homologous to the primary bacterial DNA polymerase III catalytic α-subunit (YP_009015632.1) with a DNA polymerase β (polβ) fold catalytic domain [48,49]. Together, these define four distinct replication paradigms which are outlined below. The group 1 phages are typified by a divergent DNA polymerase (e.g., YP_009153312.1) that has the apomorphic Mg^2+^-chelating DXD motif in the RRM fold β-hairpin indicating that they belong to the Pol B family (Figure 3a). These polymerases are not found outside of phages and profile-profile comparisons suggest that their closest relatives are the DNA polymerases of NCLDVs like poxviruses. However, these are characterized by certain unique features that are not found to date in any other DNA polymerases, such as an unusually long multi-helical insert just N-terminal to the 2nd strand of the core RRM fold. This insert module might compensate for the absence of a classical sliding clamp in these viruses (Figure 3a). Interestingly, these are often encoded in a conserved gene-neighborhood along with an upstream gene coding for a small SH3-fold domain protein (e.g., AAL82950.1) related to those found in certain nucleic acid-binding toxins (e.g., as 5HK3; 5XE2) [50]. It remains to be seen if these might constitute a subunit of these polymerases (Figure 3a). Of note, these DNA polymerases do not have a fused 3′→5′ exonuclease domain; however, such a protein is encoded elsewhere in the genome, suggesting that they might associate as a standalone protein in the replication complex. Going with this divergent DNA polymerase is a divergent version of the replication initiation helicase DnaB belonging to the RecA-ATP-synthetase superfamily of P-loop NTPases [51]. It is characterized by a modified ATP-binding Walker B motif. Nevertheless, its intact Mg^2+^ binding site indicates that it is catalytically active and that its divergence probably went together with that of the associated DNA polymerase.

The group 2 jumbo phages follow the basic paradigm of the coliphage T4 in having a “eukaryote-like” family B DNA polymerase as the primary replicase, which is usually accompanied by a “standard” DnaB helicase (T4 gp41) closely related to the bacterial versions. Several of these phages also code for a helicase-loader (orthologs of T4 gp59) DNA-binding protein with a domain related to the eukaryotic HMG domain chromatin proteins, which recruits DnaB to replication bubbles [52,53]. These phages are also characterized by the OB fold domain SSB (T4 gp32 orthologs), which is also usually recruited to replication sites by the helicase-loader. These phages also typically possess an AAA+ ATPase of the RFC family and its partner, the helical small subunit, which together load a sliding clamp of the PCNA superfamily on DNA [54] (Figure 3d). In the group 2.2 phages, the above family B DNA polymerase is accompanied by a remarkable, previously unrecognized, phage-specific minimal version of the DNA Polymerase III catalytic α subunit (Figure 3b). Strikingly, this version is split up into separate proteins, encoded by distantly located genes, corresponding to the N-terminal NTP-binding and C-terminal Mg^2+^- and DNA-template-binding subdomains (Figure 3b). It is conceivable that this DNA polymerase might cooperate with the more widespread family B enzyme of this group in synthesis of one of the strands or repair. The subgroup 3.1 jumbo phages show the simplest core DNA replication apparatus with the T7-like DNA polymerase as its only conserved element. The *Myoviridae* subset of these phages also displays a “standard” DnaB akin to those found in group 2 phages. A comparable *Myoviridae* subset of subgroup 3.1 also has an RFC-like DNA clamp loader but thus far lacks a detectable PCNA-like clamp homolog (Figure 3d). The subgroup 3.2 phages show a DNA polymerase III α protein with a N-terminal PHP superfamily nuclease domain. This is a likely case of non-orthologous displacement by a host-derived enzyme that they closely resemble in sequence and architecture. 

Beyond these defining elements, other DNA replication components are either more sporadic in the distribution or shared by more than one group. One such is the ATP-dependent DNA ligase that is widely represented in group 2 and a subset of group 3 jumbo phages (Figure 3c,d). On the other hand, the NAD+-dependent DNA ligase shows a complementary pattern, being primarily present in group 1, and those members of groups 2 and 3 that lack the ATP-dependent ligase (Figure 3d). Notably, most representatives of subgroup 2.1 lack any known DNA ligase. However, they code for a previously unnoticed standalone version of the α-helical DNA-binding domain typical of ATP-dependent ligases (e.g., AAX44695.1) [55] that could potentially recruit a ligase from the host (Figure 3d). Similarly, the DnaG-like primases are present in the group 2 phages and frequently in the Myoviridae subset of group 3. In the former, it also tends to be accompanied by the standalone DNA-binding primase-type Zinc-ribbon (ZnR), which is fused to the DnaG Toprim domain in the cellular primases [56]. In contrast, the Siphoviridae subgroup of group 3.1 frequently exhibits the unrelated archaeo-eukaryote-type primase (AEP) fused to an AAA+ ATPase (likely functionally equivalent to the D5 AAA+-helicase domain; Figure 3c) that can catalyze an equivalent priming reaction [57]. Notably, the group 1 phages with the divergent DNA polymerase lack both the DnaG-type Toprim domain and the AEP. Nevertheless, they all have RNase H1 protein for the removal of the RNA primer. This paradoxical situation raises the possibility that these DNA polymerases might either function as “primpols” capable of both priming [57,58,59] and elongation or that they use their multisubunit RNA polymerases for priming. Outside of group 1, RNase H1 is seen in several subgroup 2.2 phages but not any of the others (Figure 3d). However, most subgroup 2.1 and a few 2.2 phages possess a nuclease of the 5′→3′ (Flap nuclease) superfamily (e.g., APU01440) paralleling enzymes of the same superfamily found in NCLDV such as poxviruses [2,60]. This could also function as the RNA-primer degrading nuclease in these jumbo phages.

#### 3.4.2. The Core DNA Recombination, Topological Manipulation and Minor Repair Systems

Studies on medium-sized DNA viruses, such as coliphage T4, have shown an important role for multiple DNA recombination events, some of which are an essential aspect of the virus cycle. The dominant mode of replication in this virus is recombination-dependent replication (RDR), which proceeds via the invasion of a duplex by a newly synthesized single strand followed by the formation of a single-strand bubble that serves as a template that is primed by the primase-helicase complex for semi-conservative DNA replication by the viral DNA-polymerase. In T4, this homologous recombination system comprised of the ATP-dependent recombinase UvsX (the homolog of RecA/Rad51), the α-helical single strand-binding protein UvsY, the UvsW DNA helicase of the A18/UvsW family and its potential DNA-binding partner, the trihelical protein UvsW.1 [61,62]. The first three components are conserved across the group 2 jumbo phages (UvsW.1 is limited to subgroup 2.2), suggesting that they utilize the RDR mechanism. Additionally, these recombination proteins also rescue stalled replication forks along with the repair of leading-strand lesions. While UvsY and UvsW are restricted to the group 2 phages, the RecA homolog is far more widespread and can be found in the *Myoviridae* from the three groups of jumbo phages (Figure 3d). This suggests that a form of RDR is likely more widely used by jumbo phages. Consistent with this proposal, we found that both groups 1 and 3 *Myoviridae* have their own distinct versions of Snf2/Swi2 superfamily-2 helicases that could take the place of UvsW in their recombination apparatus. Subgroup 2.2 and the majority of group 3 jumbo phages also share a UvrD-type SF1 DNA helicase (T4 dda) that could also provide backup for the primary helicase UvsW in recombination [63]. It is also conceivable they have recruited their own unique single-strand binding proteins in place of UvsY. In this regard, it is notable that several group 1 and 2 phages conserve a copy of the DprA/Smf protein of the SLOG superfamily that functions as a ssDNA-binding receptor in bacteria [64], which could serve as an additional single-stand binding protein (Figure 3d).

Cellular genomes possess an alternative recombination pathway (non-homologous end joining: NHEJ) that relies on short segments of identity and depends on the DNA-end processing complex comprised of the SbcC/Rad50 ABC ATPase and SbcD/Mre11 nuclease [65]. Homologs of these are also universal in *Myoviridae* jumbo phages. Like the homologous recombination enzymes, these too are seen in numerous medium-sized phages. While they have been previously termed “repair proteins” in jumbo phages [7], given that they are retained across most representatives, they should be interpreted as part of the core recombination apparatus (Figure 3d). Like their cellular counterparts, they might be required for recombination processes related to NHEJ, such as during the bridging of phage chromosomes to mediate rescue of stalled replication forks. This might be of particular importance due to the growing knowledge of host effectors that target viral DNA potentially resulting in moribund replication forks [66]. Alternatively, these could also aid in recombination for solving the end problem, wherein chromosome ends could be lost during initiation of replication by RNA primers.

An often-under-appreciated aspect of the jumbo phage recombination apparatus is the Holliday junction resolvases (HJR). Nearly all group 1 and 3 phages possess a RuvC-like Holliday junction resolvase akin to those found in the majority of bacteria and certain NCLDV such as poxviruses [67,68]. However, as in the poxviruses, this RuvC is not coupled with a RuvB-like ATPase found in the cellular recombination systems (Figure 3d). The phage T4 endo VII-like HJR [69] that is found in subgroup 2.2 and a subset of group 4 phages shows a nearly complementary pattern with RuvC. Another, complementary association for HJRs is seen with the unrelated resolvase RusA displacing RuvC in a small subset of group 1 phages typified by the *Pseudomonas* PhikZ-like phages. That still leaves several phages without a recognized HJR. We observed that several group 2 and group 3 phages possess previously uncharacterized restriction endonuclease (REase) fold domain proteins of the RecB family (e.g., AUZ94847.1) and a novel family (e.g., APU01428.1) that are reminiscent of the REase fold HJR seen in archaea [68] (Figure 3d). Group 2.2 phages also display two conserved proteins with a Uri endonuclease domain (e.g., QAY00453.1 and APU01437.1) previously implicated in HJR function [70,71,72]. Based on contextual connections to the recombination-related single-strand coating proteins, we previously proposed a role for one of these, the T5orf172 family, in recombination [73]. It is conceivable that these endonucleases can perform an HJR-like role in phages lacking known HJRs or functions as alternative HJRs in specific repair or replication contexts.

A two-subunit phage-specific topoisomerase is found in a small subset of group 1, subgroup 2.2 and several group 3 jumbo phages. This topoisomerase forms a phage clade separate from other Toprim domain topoisomerases whose closest relatives are the cellular topo IV enzymes (Figure 3c). The phage versions are distinguished by the fusion into a single subunit of the two topoisomerase catalytic domains, namely the GHKL ATPase domain that drives conformational changes in the complex to mediate DNA-strand manipulation, and the Toprim domain that mediates strand breakage and rejoining [56]. Their second subunit is comprised of the mainly α-helical domain that forms a hoop around the single DNA strands that are moved during topological manipulation [74] (Figure 3c). The shared presence of this topoisomerase enzyme across otherwise distinct higher-order groups of phages points to the dissemination of these topoisomerases through lateral transfer between different viruses driven by the selective pressure of the independent growth in genome size. Some phages of subgroup 2.2, such as the *Achromobacter* phage Motura, Phage NCTB, *Ralstonia* phage phiRSL1, and *Pseudomonas* phages Lu11 and PaBG also possess a phage-specific version of the unrelated topoisomerase I with a tyrosine recombinase superfamily catalytic domain (Figure 3d, Supplementary Materials S1.3) [75].

Beyond these more widely conserved core components, there are additional sporadically distributed DNA repair proteins (Figure 3d). One of these is the backbone-cleaving DNA glycosylase typified by the coliphage T4 endonuclease V which is found in a subset of the subgroup 2.2 phages. It has been shown to repair pyrimidine dimer lesions [76]. In a similar vein, a subset of subgroup 2.2 and group 3 phages possesses a TIM-barrel fold UvdE endonuclease which has been shown to participate in the excision damaged nucleotides by cleaving DNA immediately adjacent to the 5′ phosphates [77].

#### 3.4.3. The Basal Transcription Apparatus and Transcription Factors

**Figure 4:**
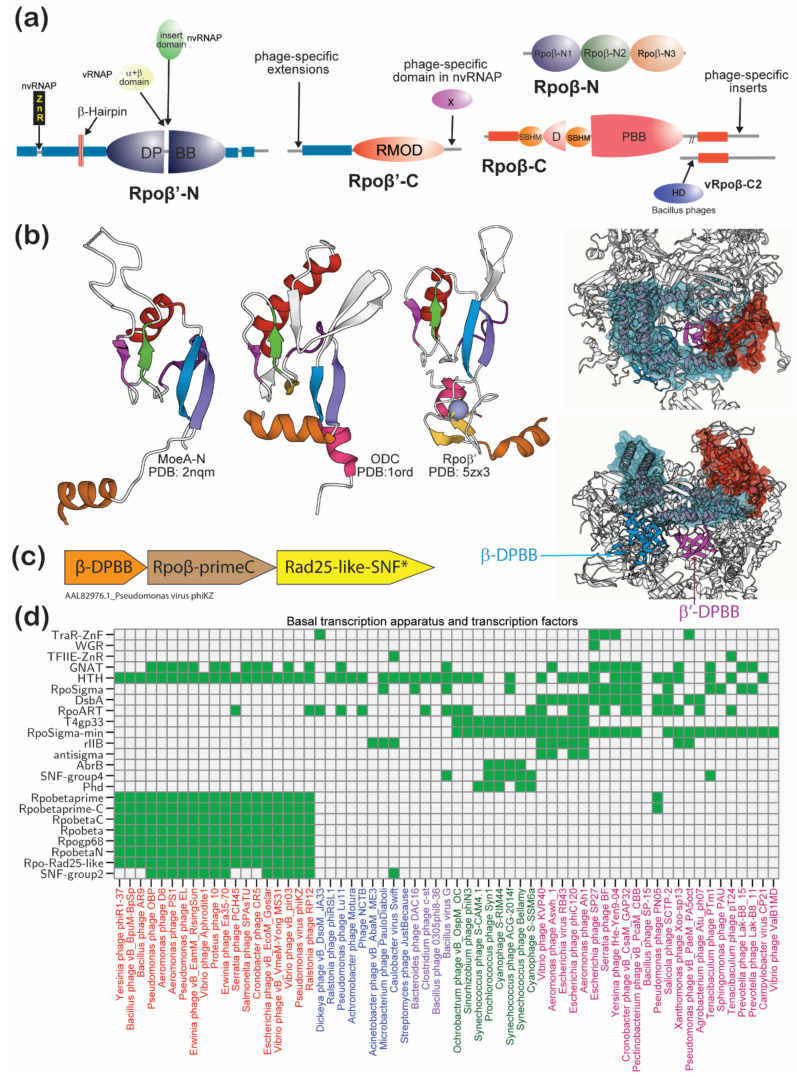
Basal transcriptional proteins and transcriptional factors in jumbo phages. (**a**) Domain architectures of the RNA polymerase β and β’ subunits found in group 1 phages. Distinct domains are colored differently. The arrows show the distinct regions of the virion (vRNAP) and nonvirion (nvRNAP) RNA polymerase-specific insertions in the universally conserved cores. (**b**) Cartoon structures of the conserved core of the RMOD domain and its location in the transcript-exit channel of the β’ subunit. On the left are MOL* cartoon renderings of the MoeA-N, the ornithine decarboxylase and the C-terminal domain of the β’ subunit and on the right are two views of the RNA polymerase, with the β’ subunit shaded blue and the RMOD domain brick red. (**c**) Gene neighborhood of the Rad25-like ATPase found in group 1 phages. The accession number in the label corresponds to the gene marked with a *. (**d**) Phyletic vector diagram of various proteins involved in transcription and transcriptional regulation. Filled green squares indicate presence of a gene. Phage names are colored by their corresponding groups. This phage order has been maintained for all vector diagrams in the figures.

Medium-sized *Caudovirales* either encode their own RNAPs, which wholly or partially transcribe their genes, or rely on hijacking the host transcription apparatus for their own transcription. The RNAPs of phages belong to either of two unrelated families defined by their catalytic domains: (1) Those with the RRM fold palm domain that are distantly related to the RRM fold palm domains of the DNA polymerases. These are found in phages like T7 or N4 and are both injected during invasion and synthesized after invasion [78]. (2) The double-barrel domain RNAPs (Figure 4a). These have an active site formed at the interface of two DPBB domains, with one supplying a triad of aspartates in a DXDXD signature that binds the two Mg^2+^ ions and the second that supplies two basic residues to stabilize the phosphotransfer reaction intermediate [17,18]. This superfamily includes all cellular and killer plasmid RNAPs, the eukaryotic RNA-dependent RNA polymerases and their caudoviral (e.g., YonO) and transposon relatives, and the archaea-specific DNA polymerases [79,80,81]. Such enzymes are also found in all the group 1 and two group 3 jumbo phages (*Bacillus* phage 0305phi8-36 and *Clostridium* phage c-st.). Those in the group 3 phages are YonO-like enzymes with both DPBB domains in a single subunit (e.g., ABS83659.1, BAE47877.1), whereas the group 1 enzymes are closer to the multisubunit host RNAPs. The latter typically come in two paralogous copies (except possibly *Pseudomonas* phage PN05 with a single β’ cognate; Supplementary Materials S1.1), one of which is packaged into the virion (vRNAP) and the other produced later for middle and late gene transcription (nvRNAP).

The jumbo phage RNAPs along with their orthologs from medium-sized phages have been extensively studied in recent works and proposed to define an ancient clade of double-barrel RNAPs [17,82]. However, as several intricacies of their architecture remain poorly described we consider those points and their significance for the evolution of this class of enzymes.

The catalytic core of cellular RNAPs consists of two subunits termed β and β’ that have accreted around the two DPBB domains [79] (Figure 4a). In group 1 jumbo phages, the equivalent of β’ is split up into two distinct polypeptides. One of these encompasses the long N-terminal α-helical array domain with a characteristic β-hairpin element and the catalytic DPBB domain that chelates the Mg^2+^ ions. The cellular β’ DPPBs are characterized by the presence of an insert domain just upstream of the active site residues, which in bacteria is an α-helical bundle domain and in archaeo-eukaryotes is a RAGNYA superfamily domain [80]. Notably, while the phage β’ DPBB also has inserts at the same position, they are neither related to the eukaryotic nor the bacterial versions. Instead, the vRNAP and the nvRNAP each have their own unique insert domains, with a longer α + β domain seen in the former (Figure 4a). Like the bacterial RNAP β’ subunit, the nvRNAPs have a Zn-ribbon inserted into their N-terminal α-helical array domain [80]. The conserved C-terminal region of β’ consists of a small 2-layered α + β domain followed by a second region, which assumes a doughnut-shaped structure that allows for the exit of the mRNA (Figure 4b). This core occurs as a standalone protein in the group 1 jumbo phages and provides hints regarding its evolutionary origins. Via structural comparisons, we established that the core of the doughnut-shaped domain is defined by a hitherto unreported superfamily (the RMOD domain; Figure 4b) that unifies the basic amino acid decarboxylase C-terminal domain and the molybdopterin biosynthesis protein MoeA N-terminal domain (DALI Z-scores 5.3–6.2; Figure 4b). In the RNAPs, this domain is inserted into a Zn-cluster domain to form a more elaborate structure that further supports α-helical hairpin extensions to form the β’ C-terminal module. The phage nvRNAP versions of this module are further distinguished by the fusion to a unique C-terminal domain that is not found in any cellular RNAP β’ subunit (Figure 4b).

The group 1 phage cognates of the cellular β subunit are split up into two separate polypeptides in the case of the nvRNAP or three in the vRNAPs. One of these corresponds to the N-terminal part of the cellular RNAP β subunit. The remainder of the β cognate is specified in the nvRNAP by a single protein containing the catalytic DPBB domain, which contributes two conserved lysines to the active site, and its C-terminal extension. The later part occurs separately as a third protein in the vRNAPs. In group 1 *Bacillus* phages, the region homologous to the C-terminal part of the cellular RNAP β subunit is further fused to an active HD phosphohydrolase domain [16] (Figure 4a). The phage β DPBB domain shares with all cellular and killer plasmid β DPBBs the insert of a sandwich-barrel-hybrid motif (SBHM) domain, which is absent in the RDRPs (and their phage relatives like YonO and NCGL1702-like RNAPs of mobile elements) [80]. The phage proteins containing the β DPBB also have a further N-terminal SBHM domain shared with the bacterial β subunits and are distinguished by a unique N-terminal region that has so far not been found in any other RNAP (Figure 4a). The SBHM inserted into the DPBB of the β subunit (the so-called “flap”) binds the basal transcription factors, either sigma or TFIIB, in the cellular versions [80]. Notably, no sigma factor is found in any group 1 phages, raising the possibility that it interacts with some other protein (Figure 4d). In the case of the nvRNAP, this could be the enigmatic phage-specific gp68 subunit. Through secondary structural analysis, we predict that this subunit is composed of a series of α-helical hairpins reminiscent of those seen in supersecondary-structure-forming domains such as tetratricopeptide and HEAT repeats. The unique inserts and domain fusions of phage RNAP subunits, which have no parallels in the cellular versions, support the idea that these phage enzymes are early-branching and have not been derived from cellular homologs via the splitting of the β and β’ subunits. Instead, they appear to be remnants of the transcription apparatus from a replicon of the pre-last universal common ancestor (LUCA) period. Hence, it is likely that such multi-subunit versions fused to give rise to the two core subunits of the cellular versions along the stem lineage leading to the LUCA.

Group 1 phages code for a Swi2/Snf2-type SF2 helicase that is specifically related to Rad25 and shows a phyletic pattern strongly correlated to the RNAP subunits (Figure 4c,d). It is also often encoded in gene-neighborhoods associated with genes for the β’ C-terminal gene and the catalytic β DPBB containing subunit (Figure 4c). Hence, this helicase might act in concert with the RNAP in transcription—one possibility is that it is a functional equivalent of the bacterial RNAP recycling SWI2/SNF2 enzyme RapA [83].

Other than group 1 and two group 3 phages, none of the remaining jumbo phages code for their own RNAPs. However, all group 2 phages code for sigma factors (Figure 4d). These sigma factors come in two types: a minimal version with just the single helix-turn-helix (HTH) domain which binds the promoter and interacts with the SBHM domain inserted into the RNAP β subunit and a more complete version close to the cellular RpoD sigma factors [80]. The minimal sigma factor seen in some group 2 phages could again be a remnant of ancient sigma factors prior to the duplication and elaboration seen in the cellular forms. The versions closer to the cellular sigma factors could have been secondarily acquired by the viruses from the cellular genomes. In addition to the *bona fide* sigma factors, the group 2 phages also possess an ortholog of the coliphage T4 late-gene transcription factor gp33 protein (Figure 4d). This protein contains a HTH domain, which recruits the RNAP by binding the SBHM domain of the β-subunit in a manner similar to the sigma and TFIIB HTH domains [84]. The sigma factors and gp33 suggest that group 2 phages adopt a strategy converse to that of the group 1 phages—they likely utilize their sigma factors to recruit the catalytic subunits of their hosts for mRNA synthesis (see below).

A neglected aspect of the jumbo phage transcription apparatus is the suite of phage-encoded transcription factors (Figure 4d). As lytic phages, it is generally believed that jumbo phages have a simple transcriptional program wherein early, middle and late genes are successively transcribed without major branching or switching for distinct alternative programs typical of lysogenic phages [85,86]. However, we found that the majority of jumbo phages (92%) code for their own specific transcription factors (TFs; median number = 2, range 1–15). While most of these have either classic HTH or the winged HTH (wHTH) DNA-binding domains, a few have the Phd and AbrB DNA-binding domains found in certain toxin-antitoxin systems [87] or the α + β WGR DNA-binding domain found in the bacterial MolR-like TFs [88] (e.g., QAY00573.1). Subgroup 3.2 phages, like *Bacillus* phage G, also show an expansion of novel Myb-type HTH TFs related to the firmicute RsfA-like TFs (Figure 4d, Supplementary Materials S1.4) [89]. At least in the case of the group 1 phages, it is possible that they deploy different TFs to work in combination with their RNAPs. The *Siphoviridae* subset of group 3 phages has an ortholog of the Zinc ribbon (ZnR) found in the archaeo-eukaryotic TFIIE (e.g., ARB13787.1) [90,91]. Given that group 3 phages lack both RNA polymerase subunits and sigma factors, it remains to be seen what role these viral TFIIE-like ZnRs might play. *Bacillus* phage G has an unprecedented lineage-specific expansion (~35 copies) of a ZnR (e.g., YP_009015343.1) that might also function as specific TFs or alternatively as structural DNA-binding proteins (Supplementary Materials S1.4). Another specific TF shared by several group 3 and group 2 phages is an ortholog of coliphage T4 rIIB protein. Early studies showed that disruption of this locus results in a more virulent phage, suggesting that it might function as a negative regulator that modulates phage transcription depending on the physiological state of the host [92,93]. In a similar vein, several group 1 (e.g., QDJ96661.1) and group 3 (e.g., ARB13751.1) phages possess repressor TFs with lambda cro/cI-type HTH domains (cHTH) that could play a role in negatively regulating certain transcriptional programs [94]. It is conceivable that the above two sets of TFs play a role in the phenomenon of pseudolysogeny reported for certain jumbo phages as a response to their hosts being under stress [44]. In principle, some of these TFs might also be used to modulate host transcription. Conversely, several group 1 phages possess predicted transcriptional activators with AraC-like HTH domains (e.g., QGZ14570.1) [95]. Subgroup 2.2 contains another potential transcriptional activator orthologous to the coliphage T4 dsDNA-binding protein DsbA that might recruit the host RNAP to late promoters [85,96]. We found that DsbA is a homolog of the *Caulobacter* GapR family of nucleoid-associated proteins [97] and is accordingly predicted to similarly form a ring around dsDNA. The above observations indicate that jumbo phages might display greater flexibility in their transcriptional programs than previously expected.

#### 3.4.4. Alternative Mechanisms of Jumbo Phages for Hijacking Host Regulatory Machinery

Studies on the coliphages T4 and N4 and more recently the *Pseudomonas* phage phiKMV have indicated that the hijacking of the host transcriptional apparatus by phages involves a battery of non-enzymatic and enzymatic factors [85]. The non-enzymatic factors include a variety of host RNAP-interacting proteins that drive it towards preferring the phage promoters. The enzymatic mechanisms include the deployment of an array of ADP-ribosyltransferases (ARTs) such as T4 Alt, ModA and ModB that modify the RNAP α-subunit and other proteins to make it switch preference for viral templates [98] (Figure 4d). Similarly, the phiKMV-like phages deploy the GCN5-like NH_2_-group acetyltransferase (GNAT) Rac to acetylate the host RNAP α-subunit leading to its eventual cleavage, thereby drawing it away from highly active host promoters to the viral early promoters [99] (Figure 4d). We recently reported the existence of both ART and GNAT domains in the large multidomain polyvalent proteins injected by certain phages into their hosts along with their DNA that might play a similar role as the above [100]. Indeed, we also found a whole slew of such factors encoded by different jumbo phages.

Among the non-catalytic hijacking factors, we found the anti-sigma factor prototyped by T4 HTH protein AsiA in a subset of group 2 jumbo phages. AsiA interacts with the primary sigma factor of the host and inhibits transcription from both bacterial and phage early promoters to cause the RNAP to switch to middle promoters [101]. We also found orthologs of the T4 Alc/Alp protein in a subset of group 2 jumbo phages (e.g., APU01786.1) that binds both the β subunit and host primary sigma factor to shut down transcription except if the DNA template contains the modified base 5hmC which is found in viral DNA (see below) [102]. Thus, it specifically redirects the RNAP for late viral transcription (Figure 4d).

Among the enzymatic modifiers, we found numerous GNATs (e.g., QBP07143.1) across all three groups that are predicted to function like the phiKMV Rac in modifying host proteins such as RNAP subunits to favor viral transcription [99]. Some GNATs found in group 1 phages (e.g., AMR59843.1) are most closely related to the ribosomal protein GNATs [103]; hence, they might help hijack the host translational apparatus or render it refractory to ribosome-targeting effectors. A comparable peptide modification is suggested by the sporadic presence of the R2K family of ATP-grasp peptide ligases in subgroup 2.2 (e.g., AUZ95380.1), a feature shared with certain amoeba-infecting NCLDVs. Members of this family of enzymes are predicted to conjugate aminoacyl moieties to translation factors in eukaryotes and could likewise aid the viruses in modifying host proteins [104]. Similarly, jumbo phages from all three groups encode several ARTs, suggesting that ADP-ribosylation of host proteins is a common strategy for mediating outcomes that are favorable to the phage (Figure 4d). We found that ARTs are most common in group 2 and group 3 phages that do not encode their own RNAPs. Therefore, at least some of these ARTs are probably used similar to the T4 enzymes in modifying the host RNAP. Also notable is the presence of the viral versions of the polyADP-ribose polymerases or transferases (PARPs/PARTs) which are typical of eukaryotes in few jumbo phages (e.g., BBA65562.1). We previously reported such versions as being shared with bacterial polymorphic toxins and being precursors of the eukaryotic PARPs [23,105]. The phage PARPs might function similarly to the ARTs but instead of a single ADP-ribose moiety transfer longer chains of ADP-ribose polymers.

**Figure 5:**
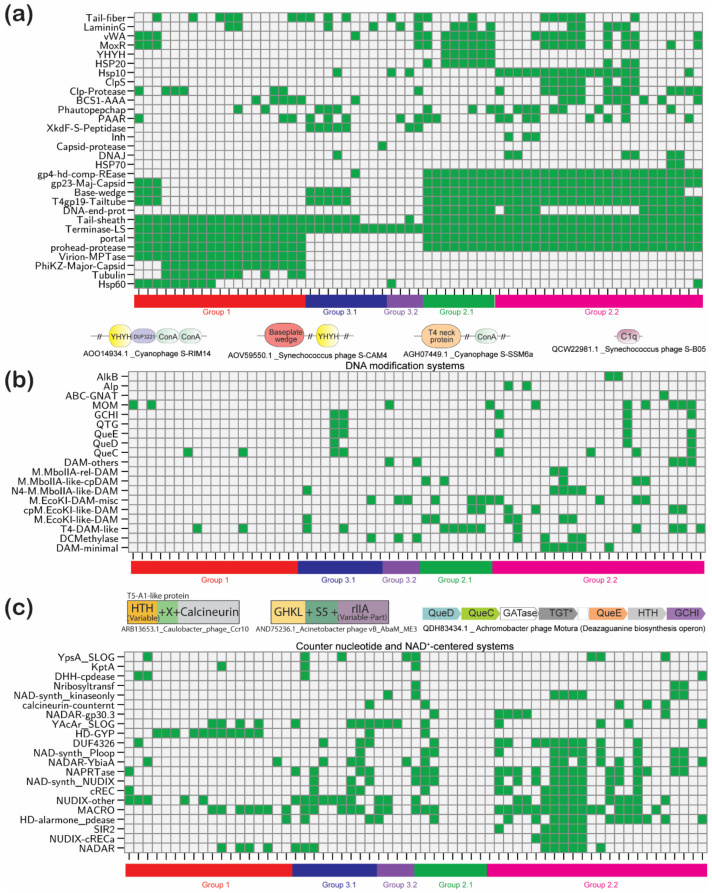
Virion structure and conflict systems. Vector diagrams, domain architectures and operons of (**a**) virion morphogenesis and chaperone proteins; (**b**) DNA modification proteins and (**c**) proteins involved in counter nucleotide and NAD^+^-centric systems. The Canberra distance and Ward clustering were used to determine protein and species order in the vector diagram. Filled green squares indicate presence of a gene. The virus name order and coloring is the same as in Figure 4. Refer to Figure 3 legend for details on domain and gene neighborhood depiction. In domain architecture representations, distinct domains are colored differently.

The observation that the acetylation by Rac triggers cleavage of the RNAP α-subunit points to the possible role of phage-encoded peptidases in targeting host proteins. Such a strategy has a much wider parallel across viruses, with multiple eukaryotic viruses targeting host ubiquitination [100,106,107,108]. Most well-conserved peptidases in jumbo phages correlate with particular virion types, suggesting that they are primarily deployed for processing viral proteins during maturation; others show a patchy pattern as might be expected of those that are deployed against the host. The most prominent of these is the ClpP serine protease (QDJ96709.1) found in some group 1 and group 2.2 phages. Several other phages instead encode their own ClpS protein (e.g., AMM43882.1), which is an adaptor that likely brings target proteins to the host ClpAP system for degradation (Figure 5a) [109]. Some group 2.2 phages also show 1–3 copies of a SprT-like metallopeptidase (e.g., APU01787.1), whereas some group 3 phages show ArdC-like MPTases [100] both of the Zincin-like superfamily (Supplementary Materials S1.6). Their limited distribution, variability, and copy number differences between closely related phages favor the possibility of them targeting different host proteins for cleavage.

#### 3.4.5. Some Notable Features of Virion Structure and Maturation

While the broad morphological features of jumbo phages and some of the conserved players in DNA packaging and virion maturation, such as the terminase and the prohead peptidases, are relatively well understood, several harder to detect or lineage-specific players in these functions remain less understood. In this section, we briefly discuss a few of these (Figure 5).

With respect to DNA-processing and packaging, the C-terminal RNase H fold endonuclease domain associated with the terminase large subunit is a universal feature across *Caudovirales* [110]. However, group 2 phages, which possess the classical terminase small subunit, are characterized by the additional virion-associated TnsA transposase-like nuclease domain with the REase fold (the so-called “head completion protein”, e.g., AMO43252.1). While this manuscript was in preparation, a report was published regarding its homolog gp4 from coliphage T4 showing that its non-specific DNase activity was required for packaging [111]. This group is also characterized by the “DNA end protector”, a HTH domain protein (e.g., AGH07633.1). This nuclease and the end protector protein likely facilitate a specific DNA-processing event required for packaging in group 2 phages. Likewise, there are several group-specific peptidases among the structural proteins that might catalyze additional processing events for virion maturation. Group 1 and 2 jumbo phages possess the portal protein (T4 gp20 ortholog) and the prohead assembly serine peptidase (e.g., AAL83076.1, coliphage T4 gp21 ortholog) that show identical phyletic patterns (Figure 5a). The latter has been demonstrated to play a role in cleavage of head proteins during assembly of the portal through which the DNA is loaded into the head [112]. Group 1 phages are distinguished by an additional conserved, uncharacterized zincin-like metallopeptidase (AMR59696.1), which could potentially catalyze other such cleavage reactions during maturation [100] keeping with reports of extensive proteolysis during maturation [113] (Figure 5a).

Some of the proteolysis events relating to the assembly and maturation of the virion are closely linked to chaperone functions that tend to have a more lineage-specific distribution. One of these is the small S74 autopeptidase domain that is found in group 2 jumbo phages (e.g., AAQ64368.1), NCLDVs of the Marseillevirus clade, and in eukaryotic DNA-binding TFs such as the myelin regulatory factor [114]. This domain is characterized by conserved serine and lysine residues that are required for catalyzing autoproteolytic detachment from the larger proteins they are part of. Subsequently, the S74 domain assembles into hexamers that aid the folding of the remainder of the parent polypeptide. This domain appears to be deployed in the assembly of tail-fiber, tail-spike and other proteins that tend to have high low complexity content [114]. Other than the Hsp10 subunit of the chaperonin complex that was noted above, Hsp20 (e.g., AUZ95449.1) was previously reported in jumbo cyanophages [115] and is also found in one of the largest jumbo phages, Atu_ph07. We found DNAJ domain cochaperones of HSP70 to be sporadically distributed among jumbo phages mainly in group 2.2 and are predicted to recruit the host HSP70 to aid folding and assembly (Figure 5a). Interestingly, group 2.2 jumbo phages also contain a previously unrecognized MoxR AAA+ ATPase (e.g., ASV44729.1)-vWA (e.g., ASV44728.2) chaperone-cochaperone pair, wherein the vWA cochaperone is fused to a N-terminal metallopeptidase domain. A MoxR-vWA chaperone system without the peptidase domain was previously reported to be required for the assembly of the tail of the *Acidianus* two-tailed archaeal virus [116]. We propose that in group 2.2 jumbo phages it similarly plays a role in virion assembly along with the peptidolytic processing of structural proteins.

Typical of most subgroup 2.2 phages is the coliphage T4 type gp40 portal assembly chaperone (e.g., APU01407.1), which indicates that in these the assembly of the prohead occurs via recruitment of the portal protein to the membrane [117,118]. A more widely distributed mechanism of membrane-proximal assembly is indicated by the orthologs of the AAA+-ATPase chaperone/translocase BCS1 that is found in several representatives of each of the 3 major groups (Figure 5a). In eukaryotes, the heptameric BCS1 protein functions as a translocase that binds fully folded proteins in the mitochondrial matrix and translocates them across the inner membrane [119]. It is possible that the phage BCS1 proteins function similarly in membrane-proximal assembly to bind and translocate folded proteins that are packaged into the head. Different subsets of subgroup 2.2 and some group 3 phages may also possess other AAA+ chaperones such as ClpA (AUZ95251.1) or ClpX (AUZ94814.1, AEO93517.1) that could play a role as translocases of unfolded proteins or aid in virion assembly (Figure 5a).

An important aspect of the mature virion is domains associated with the fibers and surface decorations of the tail and head which play a role in adhesion via specific interactions with host peptidoglycan-associated and capsular carbohydrate molecules (see below). Notable among these are different families of lectin domains that might mediate specific interactions with carbohydrate moieties on the host surface. Several of these are related to adhesion domains that are involved in cell–cell interactions in multicellular eukaryotes like animals. Such include the FGS domain of the C-type lectin fold which is found to be diversified by error-prone reverse transcriptases in several phages [120]. While we found no FGS domains in jumbo phages, previous studies have reported another β-sandwich fold lectin domain, the discoidin domain, in certain jumbo phages [121]. We found a wider distribution of this domain across group 3 phages and sporadically in other groups. We also found certain jumbo phages to contain one or more lectin domains with the concanavalin fold that are associated with the tail fibers and other virion components. These include the Laminin G3 domains and the SPRY domain (e.g., QDH50631.1) both of which have been acquired by eukaryotes. In eukaryotes, the former plays similar roles in adhesion [122], whereas the latter has been extensively used in recognition of invasive viral molecules in the TRIM-type anti-viral immune proteins [123]. Interestingly, the cyano jumbo phage *Synechococcus* phage S-B05 has a tail protein (QCW22981.1) with a C1q domain shared with the eponymous complement proteins of the vertebrate immune systems. While a domain with a similar fold was reported in the tail knob protein of the Sf6-like tailed phages [124], the above cyanophage version is much closer to the animal homologs (Figure 5a).

Several jumbo phages code for the NlpC/P60 proteins with the papain-like peptidase fold (e.g., ARB13621.1), that are sometimes tail-associated, and have been previously characterized to hydrolyze peptide-linkages in the peptidoglycan [125]. Similarly, they might also display proteins with the enigmatic YHYH domain (e.g., AOO14285.1) that is often fused to tail/base plate component proteins or the concanavalin-like lectin domains [126] (Figure 5a). We found that this domain contains a triad of highly conserved histidines and an aspartate that is predicted to form a Zn2+ binding site. Given these associations, we predict that the YHYH domains might be a hitherto uncharacterized tail-associated metal-dependent hydrolase. Thus, both the NlpC/P60 and YHYH domains appear to be a key part of the phage peptidase repertoire (in addition to the tail-associated lysozymes) to breach the peptidoglycan layer during invasion.

The PAAR domain was first found in polymorphic and related toxins delivered via the type VI secretion system (T6SS) and predicted to be an integral component of that secretion system [105]. Subsequently, it was shown to sharpen the phage-tail-like structure of the T6SS injection apparatus and also recruit toxin and other effectors to the tail [127]. The majority of *Myoviridae* jumbo phages from across all three groups possess a PAAR domain protein, which unlike those from T6SS is present in a standalone form unfused to any effector domain (e.g., APU01493.1). Thus, it represents the ancestral form of the PAAR domain found in the phage tail tip that was then repurposed as a component of the cellular T6SS.

#### 3.4.6. Conflict-Related Adaptations of Jumbo Phages

Biological conflicts between parasitic and self-sufficient replicons have shaped their genomes since the beginning of life. On the host side, this has resulted in a spectacular array of molecular immune mechanisms that limit the viral cycle by a range of different strategies. Several of these have come to light due to comparative genomic analysis and have subsequently been validated through biochemical studies [128,129]. These include disparate mechanisms such as the modification of cell-surface molecules to prevent attachment of the virus, targeting of viral nucleic acids by restriction and CRISPR/Cas systems, limiting essential metabolites such as NAD^+^ by targeted degradation, apoptotic or self-targeting mechanisms that limit viral replication and spread by targeting the ribosome or inducing cell-suicide [130]. These processes, especially those involving self-targeting or suicide, are often under tight-regulatory control by threshold-setting signals in the form of diverse nucleotides and NAD^+^ derivatives [24,131,132]. On the phage side, the counter-mechanisms against host defense and competing viruses are less understood. However, the jumbo phages are in a privileged position as they have been recently investigated for some of these mechanisms involving “fortification” of their systems by formation of subcellular compartments [133] and CRISPR-based mechanisms to hijack host functions [9]. 

Our analyses reveal that they possess several other conflict systems to counter not only the host attacks but also to prevent super-infection by other viruses and plasmids. These include: (1) an array of DNA modifications; (2) RNA repair mechanisms and provisioning of tRNAs to head off host self-attacks on the translation apparatus; (3) Metabolic systems to counter host NAD^+^ restriction; (4) Systems to target nucleotides and NAD^+^ derivatives used as signals or toxins by the host immune mechanisms; (5) Inhibitory factors to prevent superinfection; (6) Host surface modification to preclude invasion of the cell by other viruses. Beyond these, there are other poorly understood phage counter-defense systems, such as those centered on the orthologs of the coliphage T4 rIIA protein [134] which combine a N-terminal GHKL superfamily ATPase domain [62,135] with a highly variable C-terminal domain (Figure 5c). We discuss some of the major players involved in each of these processes in greater detail below.

#### 3.4.7. DNA Modifications in Jumbo Phages

DNA nucleobase modifications are among the key devices deployed across bacteriophages with DNA genomes to evade restriction attacks on their genomes by host endoDNases. These DNA modifications might also play epigenetic roles in phage-specific gene-expression and packaging of the DNA into the head [38,136,137]. Further, modification of phage DNA enables them to launch endonucleolytic attacks on the host DNA to preempt expression of host-counter phage defenses and potentially recycle host nucleotides for phage replication [138]. DNA modifications in jumbo phages came to light with the *Sphingomonas* phage PAU whose genome size was misestimated by certain electrophoretic procedures due to its extensive DNA modifications [139]. It was also observed that multiple *Bacillus* jumbo phages have almost all thymine in their DNA replaced by uracil [140,141]. Our systematic analysis of the jumbo phage genomes reveals that all three major mechanisms of DNA modifications can be seen in different lineages: (1) production of pre-modified nucleotides, like UTP or hydroxymethyl CTP, for DNA synthesis; (2) in situ modification by specialized DNA modification; (3) production of a pre-modified base followed by its incorporation in DNA via a transglycosylation reaction.

In terms of the phages using pre-modified nucleotides for DNA synthesis, multiple negative determinants are required for using uracil in place of thymine, namely the absence of a thymidylate synthase or a Thy1, and a dUTPase (Supplementary Materials S1.1, S1.5). This unique situation is seen in the *Bacillus* jumbo phages like PBS1 that use uracil in place of thymine [16]. Phages with specialized thymidylate synthase superfamily enzymes for synthesis of 5 hmC can generate that base in a manner similar to the synthesis of thymine from uracil [38]. Other than the dUTPase, there are also MazG-like and CYTH superfamily triphosphatases scattered across all groups of jumbo phages, which could potentially play a comparable role in degrading endogenous NTPases with unmodified nucleobases (Supplementary Materials S1.1, S1.11). A subset of these phages often has a second determinant in the form of an ortholog of the T4 Alc protein that helps redirecting the host RNA polymerase solely towards templates that contain 5 hmC [102] (Figure 5b). This is primarily seen in a subset of group 2.2 phages. The absence of TET/JBP pyrimidine hydroxylases suggests that all 5 hmC in jumbo phages characterized to date is synthesized at the level of free nucleotide and not generated in situ in DNA [136].

We extend the previous observations of DNA N^6^ adenine methylases (DAMs) and DNA 5-cytosine methylases (DCMs) in jumbo phages to define several previously unrecognized exemplars of these [137] (Figure 5b). The DAMs can be classified into three higher-order clades and the related cytosine N4-methylases; representatives of all these are found in different jumbo phages. The most common are the coliphage T4-like DAMs (Clade 3) which are found in representatives of all three higher-order groups of jumbo phages (e.g., AUZ94846.1). The next most prevalent are the M.EcoKI-like DAMs (Clade 2), a circularly permuted version of it and other smaller related clades are found in some group 2 and 3 jumbo phages (e.g.,APU01582.1). Similarly, some group 2 and 3 phages also possess the M.MboIIA/M.MunI-like DAMs (Clade 1) with or without a circular permutation (e.g., AUZ95158.1, AQW88654.1). A subset (AAX44419.1) of these methylases shows the characteristic motifs that indicate them as being cytosine N4-methylases [137] and are found sporadically in members of groups 2 and 3. A distinct family of DAMs from subgroup 2.2 (AMM43637.1), which are much smaller than the DAMs with DNA substrates, are closest to the Clade 3 DAMs defined by the PCIF1 enzymes, which were recently shown to methylate adenines at the 5′ ends of eukaryotic mRNAs [137,142]. Hence, these phage enzymes might also be comparable RNA adenine methylases that play a role in post-transcription regulation. Lastly, DCMs are also found in group 2 and group 3 phages. A small number of phages display an AlkB-like 2OGFeDO (e.g., AXQ68776.1) which acts on methylated adenines either in the context of DNA repair or resetting of the methyl marks [143,144]. Its sporadic and limited presence suggests that rather than DNA repair it might be directed at resetting methyl marks either in the phage as part of an epigenetic regulatory process or in host DNA to target protective host DNA adenine methylation (Figure 5b).

Other than these well-known modifications, we also identified enzymes for other larger DNA-modifications that have previously not been reported in jumbo phages. Several group 2 phages code for enzymes of the Mom family of the GNAT superfamily that are prototyped by the Mom enzyme of phage Mu. This enzyme catalyzes the transfer of an acyl group from an acylated coA moiety, such as glycyl coA, to the N^6^ atom of adenine (Momylation) [145,146]. Since then, several momylation systems are encoded by both phage and bacterial genomes [136,145]. The jumbo phage Mom domains are typically fused to REase fold endoDNase domains that are predicted to function as the restriction component (Figure 5b). This implies that Momylation protects the phage DNA via modified adenines, while the associated endonuclease domain attacks the DNA of the host or superinfecting phages. We previously identified a further member of the GNAT superfamily coupled with an ABC ATPase domain that was predicted to carry out a yet uncharacterized acyl modification of a DNA nucleobase [136]. This DNA modification system is found in a small subset of group 2 jumbo phages such as the *Tenacibaculum* phage pT24 (e.g., QAX98348.1) pointing to hitherto unrecognized modifications among the jumbo phages (Figure 5b). Similarly, a small number of jumbo phages such as the *Achromobacter* phage Motura contain a system for the synthesis of a deazaguanine base like queuine or archaeosine, and a transglycosylase enzyme for their incorporation into DNA in place of guanine [136] (Figure 5c). In some jumbo phages, only a subset of enzymes of this system is observed (Figure 5b). However, given that several bacteria possess such DNA modification systems, it is possible that the phages complement their deazaguanine production and incorporation systems with the host enzymes [136].

DNA modification enzymes are most common in group 2 and 3 jumbo phages (Figure 5b). These might contain more than one such system—for example, phage vB_PaeM_PA5oct contains five momylating enzymes and a deazaguanine synthesis/incorporation system (Supplementary Materials S1.11 and S5). Similarly, multiple phages might possess more than one type of DNA methylase. This multiplicity possibly helps them overcome more than one host restriction system. The group 1 phages generally possess fewer DNA modification systems, keeping with the proposal that the nucleus-like compartment formed by them limits host restriction attacks [19,20] (Figure 5b). However, those that do carry such enzymes could utilize them for protecting viral DNA immediately after invasion.

#### 3.4.8. Counter-Nucleotide and NAD^+^-Centered Systems

A key discovery in recent years is the pervasive role of cyclic and linear (oligo)nucleotides, nucleotide- and NAD^+^ derivatives as signals in immune mechanisms across the three superkingdoms of life [24,131,147,148,149,150,151]. Such signaling molecules are produced by dedicated signal-generating nucleotide synthetases, the Ter-biosynthetic system (see below), and NAD^+^-processing enzymes and induce a diverse array of effectors that target the viral or host macromolecules (in a suicidal or dormancy-inducing response) [24,131,152]. The systems which are united under the umbrella of these signaling mechanisms include the eukaryotic and prokaryotic SMODs (e.g., interferon-induced oligoadenylate synthetase and cyclic guanylate-adenylate systems), the prokaryotic type-I and type-III CRISPR/Cas, the SLOG-TIR and alarmone-based immune systems [131]. There is increasing evidence, especially in eukaryotic viruses, that there are several viral counter-mechanisms that degrade the nucleotide/nucleotide-derived signals to block these immune processes [153,154]. The analysis of the polyvalent proteins injected by certain tailed phages along with their DNA suggested that such signal-targeting enzymes might be widespread even among bacterial viruses [100]. Here, we present the identification of several such proteins across jumbo phages that provide prototypes for understanding such counter-nucleotide defenses in the viral world. These further enmesh with various viral NAD^+^-centered systems.

**Figure 6:**
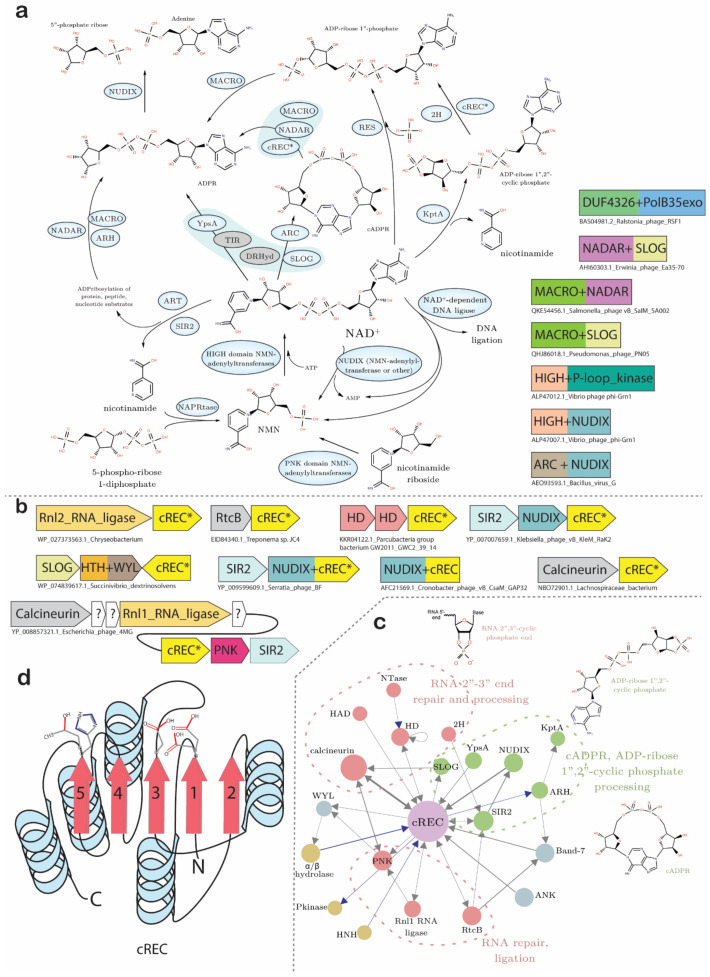
NAD^+^-centric conflict systems. (**a**) Biochemical action of various proteins involved in the NAD^+^-centric conflict systems and examples of interesting domain architectures of these proteins. (**b**) Gene neighborhoods of the cREC-containing systems and predicted topology of the cREC domain. Genes are represented as block arrows with arrow-heads pointing to the 3′ ends of the gene. Neighborhoods and architectures are denoted by their accession numbers and phage names separated by underscores. The accession number in the label corresponds to the gene marked with a *. (**c**) Contextual network of the cREC domain derived from gene neighborhoods and domain architectures. Gene neighborhood associations are depicted with black lines with the arrowhead pointing to the 3′ gene and domain associations with blue lines with the arrowhead pointing to the C-terminal domain. Distinct domains in architecture representations are colored differently. Nodes in the same functional category are colored similarly and grouped. (**d**) The topology of the cREC domain was predicted based on the know Receiver domains. The conserved residues were then superimposed onto the topology based on the cognate positions of conserved residues observed in classical Receiver domains.

We found at least six distinct families of phosphodiesterases (PDease) that we predict to be the mainstay of counter-nucleotide defenses in jumbo phages, which are likely directed at different types of nucleotides (Figure 5c). Across the three jumbo phage groups, there are HD-GYP enzymes (e.g., QDB70412.1) of the HD superfamily of phosphoesterases that target cyclic di- and oligo-nucleotides [155,156,157,158] and are likely to serve as a defense against the SMODS-based and type I/III CRISPR systems that use such nucleotides as signals. Another member of the HD superfamily, HD-alarmone PDease from group 2 and 3 jumbo phages (e.g., BBI90576.1), likely targets the alarmone [159], a nucleotide used as the signal by the stringent response system and the recently described related host immune systems [131,160]. PDEases of the DHH superfamily, which have been shown to act on diverse cyclic linkages, such as 3′-5′, 2′-3′ and cyclic di-nucleotides [161,162,163], are also found in a small number of group 2 and 3 phages (e.g., ALN97901.1) and possibly counter signals of SMODS and type I/III CRISPR systems [164]. A similar activity is predicted for the coliphage T5 A1 orthologs [165], which are phosphodiesterases of the calcineurin-like superfamily [48], featured by a set of group 2 and 3 phages. Members of the 2H superfamily of phosphoesterases found in group 2.2 and some group 3 jumbo phages (e.g., QDH83605.1) are specialists of 2′3′ cyclic phosphates that are formed from the cleavage of tRNA and NAD^+^-dependent RNA processing (Figure 5c and Figure 6a) [166,167]. These could signal the attack on the translation apparatus and potentially induce other defense systems. Phage enzymes targeting such cyclic phosphates could play an additional role in RNA repair (see below).

NAD^+^ is used as a substrate by enzymes of the ART, SIR2, ADP-ribosyl cyclase (ARC), TIR and SLOG superfamilies to produce a variety of ADPr derivatives, which are either conjugated to other macromolecules (ADP ribosylation), constitute soluble signals such as ADPr 1′′2′′ cyclic phosphate or cADPr with a cyclic diphosphate linkage or potential toxic metabolites such as ADPr 1′′phosphate [24,131,168,169,170,171,172,173,174] (Figure 6a). Soluble ADPr-derived signals are central to the counter-phage defense systems that feature domains belonging to the above superfamilies. Such host-generated soluble ADPr-derivative signals (e.g., cADPr) and toxins (ADPr 1′′P) are potentially degraded by the Macro (e.g., APU01542.1) and NADAR (e.g., QAY00367.1) enzymatic domains, which are displayed by several jumbo phages across all three groups and shared with unrelated families of eukaryotic RNA viruses [23,175,176]. A subset of these might also function in NAD^+^-dependent RNA repair (see below). Through the analysis of conserved gene-neighborhood or operonic linkages, we uncovered a divergent version of the Receiver (Rec) domain that is linked to different NAD^+^/ADPr-processing enzymes (Figure 6b–d). Unlike conventional Rec domains, this domain is never found in two-component Histidine Kinase signaling systems [177]. Instead, it is found only in association with NAD^+^-processing systems that generate or recognize cyclic nucleotides (e.g., 2′-3′ cyclic ends of RNA and WYL domains that recognize cyclic nucleotides [178,179]; Figure 6b). Known Rec domains process phosphoester linkages by means of catalytic aspartate residues that are also conserved in these divergent Rec domains [180]. Accordingly, we posit that these are cADPr or 2′-3′ cyclic nucleotide-processing enzymes and term them the cRec (cyclic-phosphate processing Rec) domains. The linearized ADPr generated by the Macro, NADAR and cRec domains or by NADase action of host NAD^+^-targeting effectors (see below) are likely to be processed further in certain group 2 and 3 phages by the Nudix domain (e.g., QDH83582.1), which specifically cleaves nucleotide diphosphate-X linkages, to release AMP [181,182] (Figure 6a,c). 

On the phage side, too various systems such as replication (NAD^+^-dependent DNA ligases) and RNA repair [105,176,183] use NAD^+^ as an essential substrate. Moreover, ADPr conjugates to proteins catalyzed by the ARTs (see above) are also a key part of the phage control of the host machinery. Some jumbo phages possess SIR2 (e.g., QAY00582.1) and YAcAr family SLOG domains (e.g., QBP07259.1). We also detected novel members of the ARC superfamily in certain jumbo phages belonging to groups 2 and 3 (e.g., AEO93593.1). All these domains are known or predicted to utilize NAD^+^ [131,184] to produce ADPr-derivative signals, such as cADPr. These domains are frequently fused to MACRO, NADAR, or Nudix domains in the same polypeptide (Figure 6), which can degrade these signals, suggesting that they could act as toggles deployed by the jumbo phages to control host behavior using ADPr-derivative signals. Our contextual analysis of domain architectures also revealed the hitherto enigmatic domain DUF4326 (Figure 5c and Figure 6a) found in several group 2 and 3 jumbo phages as well as amoeba-infecting NCLDVs to show several independent fusions to Macro, SLOG and NADAR domains on one hand and DNA replication or R-M components on the other. Accordingly, we predict it to be a potential enzymatic domain that bridges NAD^+^-utilizing signaling systems with DNA-modifications that interface with replication and R-M systems (AMB and LA, in preparation).

Host defense systems counter the viral requirement for NAD^+^ by the deployment of NADase effectors, such as the TIR, DrHyd and SIR2 domain proteins [24,131,168,169,170,171,172,173]. Given that NAD^+^ limitation can cripple all the above-mentioned systems, it is not surprising that several jumbo phages have been previously reported to carry their own NAD^+^-synthesis system [21]. The current analysis reveals that this system exists in certain representatives of each of the three major groups of jumbo phages (Figure 6a). The most expanded version found in phages such as *Vibrio* phage phi-Grn1 which has 3 genes: a nicotinamide phosphoribosyltransferase (NAPRtase; e.g., ALP46980.1) [185] and two distinct nicotinamide-nucleotide adenylyltransferase genes. The first enzyme synthesizes nicotinamide mononucleotide NMN from nicotinamide and 5-phospho-ribose 1-diphosphate. Both versions of the adenylyltransferases contain a HIGH superfamily NTase domain that adenylates NMN. However, they are differentiated by fusions of the HIGH NTase domain to either a Nudix domain (e.g., ALP47007.1), which can hydrolyze NAD^+^ to NMN [186], thereby regulating NAD^+^ levels or a P-loop kinase domain (e.g., ALP47012.1), which phosphorylates nicotinamide riboside to generate NMN [187]. Some jumbo phages contain more abbreviated versions of these systems with a single NAPRtase-NTase pair or just the former enzyme.

#### 3.4.9. RNA Repair and RNA-Based Regulatory Systems

**Figure 7:**
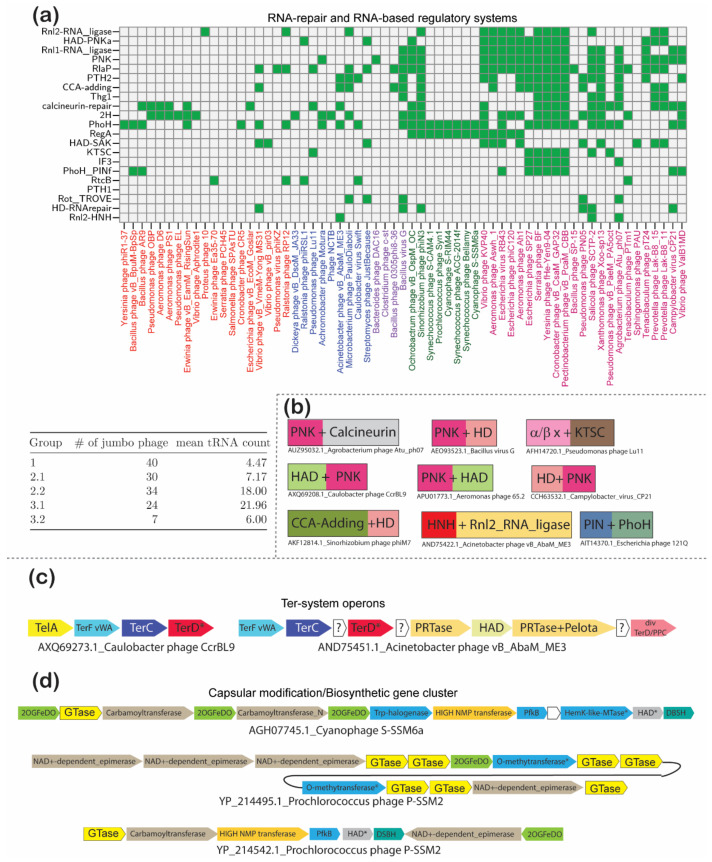
RNA-repair and RNA-based regulatory systems. (**a**) Phyletic vector diagrams and (**b**) domain architectures of jumbo proteins predicted to be involved in RNA-repair and RNA-based conflict and regulation. Also shown in (**a**) is the mean tRNA count for the different groups of jumbo phages. Also illustrated are the gene neighborhoods of (**c**) Ter-system and (**d**) capsular polysaccharide modification systems. GTase: glycosyltransferase. Genes are represented as boxed arrows with arrow-heads pointing to the 3′ ends of the gene. Neighborhoods and architectures are denoted by their accession numbers and phage names separated by underscores. The accession number in the label corresponds to the gene marked with a *. In domain architectures, distinct domains are colored differently.

Bacteria possess several “second-line” immune mechanisms that are brought into play when the primary invader-targeting restriction mechanisms fail [24,131,188]. Typically, these restrict phage replication by targeting their own translation system components, such as tRNAs and ribosomal proteins. A widespread mode of attack is in situ cleavage of the tRNA anticodon loops at the ribosome to “jam” translation [189,190,191]. While these measures might negatively impact the host fitness, in the net they prove beneficial because they can limit the spread of the virus to kin cells (inclusive fitness) and the host could potentially tide over periods of dormancy while stopping the viral cycle. Starting with the work on the coliphage T4 [192,193], it became clear that there are several RNA repair/ribosome rescue processes by which phages reverse such attacks on the translation system [178]. The best-described of these is the presence of phage tRNAs in the genomes of disparate jumbo phages [9,194,195,196], which could substitute for the cleaved host tRNAs to allow translation of viral proteins to continue. We found that group 1, and subgroup 2.1 jumbo phages on an average possess only 4.5 and 7.1 tRNAs, respectively, per genome. However, subgroups 2.2 and 3.1 possess a mean number of 18 and 22 tRNA per genome, respectively (Figure 7a). This suggests that the reliance on self-encoded tRNAs widely differs between phages. More generally, we found RNA repair systems to be more common in group 2 and 3 than group 1 phages (Figure 7a). This difference presumably arises from the protection offered by their nucleus-like compartment.

In all three jumbo phage groups, the tRNAs are supplemented by two classes of RNA ligases that ligate cleaved tRNAs, which belong to the RtcB-like and the ATP-grasp folds, respectively [178] (Figure 7a). The RtcB ligases are less common in jumbo phages and ligate RNAs both with 3′ phosphate and 2′-3′ cyclic phosphate ends, which are generated by the action of the metal-independent RNases [197,198] (Figure 7a). In contrast, the ATP-grasp ligases [199,200], which are more common in jumbo phages, require RNA ends with 5′ phosphates and free 3′ hydroxyls as substrates [201]. The majority of phage ATP-grasp ligases belong to one of two families, Rnl1 and Rnl2, with some rare divergent versions, like those from actinophages (e.g., AYD81305.1), which are outside of these families. Certain members of the Rnl2 family are fused to N-terminal HNH endonuclease domains [178] that might be used to launch attacks on superinfecting phage or host DNA even as the ligase repairs damaged RNA. A given phage might have any combination of RtcB, Rnl1 and Rnl2; e.g., the *Burkholderia* phages BcepSaruman and BcepSauron have all three of them, whereas *Xanthomonas* phage XacN1 has three paralogous versions of the Rnl2 family (Figure 7a, Supplementary Materials S1.8 and S5). Hence, these RNA ligases are probably not redundant and have specialized roles in repairing RNA cleaved by different kinds of RNase effectors. A small subset of group 3 and 2 phages additionally feature a ROT/TROVE RNA-binding protein, which, in cellular organisms constitutes a ribonucleoprotein platform for RNA repair along with a tRNA-like partner [178,202] (Figure 7a).

As several RNase effectors, especially the metal-independent RNases, generate RNA ends that are not directly usable by ATP-grasp ligases, they function along with polynucleotide kinases which phosphorylate the 5′ ends, and phosphoesterases which hydrolyze 3′ phosphates and make them amenable to ligation [178]. In jumbo phages, such enzymes are generally congruent with the presence of ATP-grasp RNA ligases (Figure 7a). The clearing of cyclic 2′-3′ ends for ligation by ATP-grasp RNA ligases is likely performed in part by the above-mentioned 2H phosphoesterases that are specialist enzymes for such linkages [166,167]. Additionally, as noted above, the cRec phosphoesterase domain is likely to perform such a role in a subset of the jumbo phages. In support of this proposal, we found several operonic linkages of the cRec domain to the different families of RNA ligases in both phage and cellular genomes (Figure 6b). After the cyclic ends are resolved, the linear phosphates are hydrolyzed by linear phosphatases. In jumbo phages, these are typically members of the HAD superfamily and these belong to either of two families: PKN-HAD (e.g., QAX98520.1) usually fused to a N-terminal polynucleotide kinase domain and the so-called “Swiss-army-knife” (e.g., AUE22617.1) family which [178] tends to occur as a standalone domain (Figure 7b). In a small number of jumbo phages, this phosphatase activity appears to be also catalyzed by calcineurin-like and HD-like domains. In few group 1 and 3 jumbo phages, the 2′ phosphates emerging from the action of metal-independent RNases might also be “cleaned up” by the action of the KptA family of ARTs (e.g., AUG85871.1) that transfer the phosphate to NAD^+^-generating a 1′′-2′′cyclic phosphorylated ADPr [23,203], which is further processed by 2H and Macro domains [204] (Figure 7). 

Other than ligating RNAs targeted by endoRNases, jumbo phages deploy one or more template-independent and template-dependent nucleotidyltransferases (NTases) to restore missing bases from RNA ends. The most prevalent of these is the recently described RlaP (RNA ligase-associating Polβ) NTase family that functions alongside the RNA ligases [178]. Its distribution in jumbo phages closely mirrors the RNA ligases supporting the functional cooperation between these enzymes (Figure 7a). RNase effectors deployed by host immune mechanisms, in addition to endonucleolytic action, might exonucleolytically cleave off a further nucleotide from the end (e.g., RloC RNase action on the wobble base after cleaving the anticodon loop [205]). It is predicted that the RlaP NTases restore such lost nucleotides before ligation [178]. The next most common is the CCA-adding enzyme (also of the Polβ superfamily) found in several group 2 and 3 jumbo phages, which restores the CCA trinucleotide or parts thereof in a template-independent manner at the 3′ end of tRNAs [206]. In a similar vein, nucleotide restoration at the 5′ ends (especially in the histidinyl tRNA) is catalyzed by the unusual 3′-5′ polymerase Thg1 in a template-dependent or independent manner [207]. This enzyme is only sporadically present in a few group 2 and 3 jumbo phages (Figure 7a).

Cloven tRNAs at the ribosome pose an additional challenge—they are linked to the incomplete polypeptides through a peptidyl tRNA linkage. Hence, hydrolysis of such peptidyl tRNA linkages is an important step in the recycling process to rescue jammed ribosomes [208]. Across group 2 and 3 jumbo phages, we found the peptidyl tRNA hydrolase (PTH2, e.g., APU01446.1) [195] which can potentially hydrolyze such linkages during ribosome rescue (Figure 7a). Interestingly, PTH2 is the characteristic peptidyl tRNA hydrolase of the archaeo-eukaryotic lineage [209] rather than that of host bacteria, which instead usually contain Pth1; thus, it could be the remnant of the translation apparatus of an ancient replicon that contributed to both archaeo-eukaryotic and phage genomes. In contrast, a single group 2 jumbo phage identified from metagenomic data with a bacterial-type Pth1 (AXH72886.1) [195]. The translation initiation factor IF3 gene (e.g., AUZ94813.1) and the lysyl tRNA synthetase KTSC RNA-binding domain protein (e.g., AZU98290.1) seen in few group 2.2 phages could also play a role in ribosome rescue or supporting phage translation (Figure 7a).

A further RNA-related function found across several group 2 and 3 phages is the PhoH protein, which contains an ATPase domain related to the N-terminal domain of SF1 helicases [210]. PhoH proteins have been shown to function as RNA helicases and at least some of these are sequence-specific. Both in jumbo phages and cellular genomes, they might be coupled to a N-terminal PIN endoRNase domain with the 5′→3′ nuclease fold [87]. This combination has been shown to function in cellular genomes as a toxin-antitoxin system that potentially facilitates dormancy in response to certain environmental stresses [210,211]. Its role in the jumbo phages remains enigmatic; among other roles, these could help in the clearing of jammed ribosomes via its helicase action and endonucleolytic action of the associated PIN domains when present. Alternatively, it could function in post-transcriptionally regulating certain genes by operating on their RNAs. This latter function is related to the proposed role in post-transcription gene regulation for the orthologs of the coliphage T4 RegA proteins with a RRM fold RNA-binding domain [212] that are found across group 2.1 jumbo phages (Figure 7a). The presence of a conserved histidine in these proteins raises the possibility that RegA might function as a metal-independent RNase (Supplementary Materials S1.8 and S5).

#### 3.4.10. Pseudolysogeny and Adaptations for Preventing Superinfection in Jumbo Phages

In a subset of jumbo phages, specialized tubulin/FtsZ-like proteins facilitate their chromosome segregation synchronously with host cell division during pseudolysogeny [213,214]. There is evidence that at least some of the jumbo phages without tubulin homologs (e.g., cyanophages) are also capable of pseudolysogeny during which they alter host-biochemistry, such as the photosynthetic apparatus. In addition to attacks from the host immune systems, jumbo phages also experience heightened conflict during pseudolysogeny with other phages and plasmids that might invade the cells they are residing in. Other than deploying the R-M systems [213,214] and CRISPR systems [213,214] encoded in the phage genomes, several jumbo phages have evolved at least two alternative strategies for these conflicts.

The first strategy involves the deployment of intracellular inhibitory factors that prevent the establishment of rival phages. We observed that several jumbo phages possess one or more proteins from the Ter system (Supplementary Materials S5), a functionally linked network of proteins found across bacteria that is involved in resistance to phages and certain xenobiotic substances [152]. The Ter system is also encoded by some plasmids which use it as a “fertility inhibitory factor”, i.e., preventing other plasmids from parasitically utilizing their transfer apparatus [215]. The Ter system includes several functionally disparate components: (1) biosynthesis of a nucleoside/nucleotide derivatives (nucleobase phosphoribosyltransferases: PRTases and HAD fold phosphoesterases), which evidently signal the detection of the incoming threat; (2) ligand-binding domains such as the TerB and TerD proteins that might sense the signal; (3) TM components that might respond directly by changing membrane structure and transport (TerC); (4) components forming sub-membrane structures (e.g., TelA and the TerF vWA domain protein) which prevent entry of the phage or xenobiotic [152]. We found a relatively complete Ter system with signal biosynthesis PRTases and HAD enzymes in the group 2.2 jumbo phage, *Acinetobacter* phage vB_AbaM_ME3 (Figure 7c). More abbreviated Ter operons with TerD, TerC, TerF and TelA are seen in the group 3 *Caulobacter* jumbo phages. As standalone genes, TelA is also found in the gammaproteobacterial group 2.2 jumbo phages, TerB in group 1 *Erwinia* phages (e.g., ANZ49651) and TerD in actinobacterial group 3 phages. These observations suggest that the jumbo phages (and certain other related smaller tailedphages), might utilize the Ter system in their competition with superinfecting phages. Additionally, these genes might also confer a degree of xenobiotic resistance to the host, thereby allowing the phage to tide periods of pseudolysogeny.

The second mechanism is to modify the host biochemistry. This includes modification of molecules such as the capsules and peptidoglycan to prevent to counter attachment of rival phages [22]. Bacteria with Gram-negative cell walls are characterized by a complex glycolipid component of the outer membranes, the lipopolysaccharide (LPS), comprised of less-variable lipid A and oligosaccharide components and the highly variable outward-facing polysaccharide (O antigen) component. In contrast, Gram-positive bacteria have their own surface structures such as teichoic acid and teichuronopeptide shells. Systems modifying surface molecules have previously been found as part of the cellular defense against viruses [130] and have recently been reported to be coupled to intracellular counter-viral defenses to provide a two-pronged mode of immunity [24]. While these systems have been long recognized in lysogenic *Caudovirales*, the presence of such systems in jumbo phages first came to light with the report of the so-called LPS biosynthesis system in group 2.1 cyanophages [7]. Our analysis points out that such systems might more widespread and sometimes more elaborate than originally reported.

*The Synechococcus* phage P-SSM2, the prototypical group 2.1 jumbo phage with such systems, has a major 15-gene cluster. Such large clusters are primarily seen in cyanophages [7] (Figure 7d), with smaller complements of modifying enzymes in some of the other group 2 and 3 phages [22]. The cyanophages display complete complements of sugar-production and modification enzymes, whereas the other jumbo phages have more restricted sets of enzymes. The complete clusters contain 1–4 paralogous genes coding for a NAD^+^-dependent epimerase/dehydratase that are key enzymes in the production of modified sugars (Figure 7d). They may also feature the double-stranded β-helix (cupin) fold sugar isomerases [216]. The presence of PfkB family sugar kinases [217] in the cyanophages suggests that these catalyze the production of phospho-sugars (Figure 7d). These act as the substrates for the production of NDP-linked sugars by the HIGH-superfamily nucleotidyltransferases that add NMP to phosphosugars (e.g., QAY00630.1, AGN12290.1) [218], which have a wider distribution beyond the cyanophages. The mainstay of these clusters is one or more paralogous glycosyltransferase (GTase) genes belonging to one of at least eight distinct GTase families [219] that synthesize oligosaccharides using NDP-sugars as substrates (Figure 7d). Their sporadic presence and polymorphisms seen in the cyanophage gene-clusters even between related viruses suggest that their products are primarily involved in the production of diversified outer polysaccharide complements (O antigen in the case of LPS) that might aid in preempting attachment of other phages.

We observed that cyanophages might feature a second gene cluster with 8–12 genes coding for sugar- and amino acid-modification enzymes. For instance, jumbo cyanophages like S-SSM6a contain a 10 gene cluster that is predicted to specify a biosynthetic system producing a halogenated amino acid and sugar-derived secondary metabolite (Figure 7d). The core of this system shared with actinobacterial secondary metabolite biosynthetic systems features carbamoyl transferases (e.g., AGH07737.1, AGH07739.1) related to those found in beta-lactam antibiotic synthesis and a HemK-like amino acid methylase (e.g., AGH07745.1). The cyanophage gene-cluster also codes for an amino acid halogenase (AGH07741.1), several hydroxylating enzymes of the 2OGFeDO (e.g., AGH07740.1) and cupin-like DSBH (e.g., AGH07747.1) families and sugar-dephosphorylating HAD superfamily phosphatases [220]. Variations on this theme might include additional O-methyltransferases (e.g., YP_214498.1, YP_214495.1; Figure 7d). This system functions either in the further decoration of capsular polysaccharides or in producing a secondary metabolite and might serve as an antibiotic or toxin to improve host competitiveness during pseudolysogeny.

### 3.5. Evolutionary Considerations

Jumbo phages provide a window into understanding key evolutionary questions: (1) What was the form of the proteins performing key functions encoded by early replicons? (2) What were the evolutionary events that shaped the domain architectures of proteins associated with information transmission through the “central dogma” prior to the last common universal ancestor (LUCA) of cellular life? (3) How did the early replicons accrete genes to become larger replicons with increasing degrees of self-sufficiency?

Since the availability of the first viral genome sequences, it has been discussed as to whether they carry any features of early replicons beyond those that can be inferred from cellular life forms or if they represent different stages of the “degeneration” of cellular life [221]. The jumbo phages on the bacterial side and the NCLDV on the eukaryotic side have been at the center of this discussion as their large genomes straddle the size range between the smallest cellular genomes and medium-sized viral genomes [2,3]. In the past three decades, the availability of degenerate cellular genomes, such as *Mycoplasma genitalium*, the bacteroidetes *Sulcia muelleri* and the gammaproteobacterium *Baumannia cicadellinicola*, has emphatically indicated that they display gene complements and phylogenetic relationships quite unlike the phage proteins [222,223]. First, despite extreme degeneration, these bacteria retain sufficiently large complements of “core proteins” (i.e., those associated with central dogma information flow: replication-, transcription- and translation-related proteins). Second, these proteins show high sequence similarity with those of other bacteria, usually sufficient to precisely position them on the bacterial phylogenetic tree. Third, the conserved proteins show mostly congruent domain architectures to their bacterial counterparts.

In sharp contrast, jumbo phages do not show any tendency to conserve relatively complete complements of core systems. Instead, they possess a patchwork of such in so far as they help their successful replication. Thus, jumbo phages despite their size, might entirely lack an RNAP and usually do not code for a conserved aminoacyl tRNA synthetase complement, which is found even in the highly degenerate bacterial genomes. Further, the core systems that are present can be markedly divergent from all their host counterparts—the divergent DnaB and family B DNA polymerases typical of the group 1 jumbo phages are a case-in-point. This has also been demonstrated for the multisubunit RNA polymerases [16,17,18]. Likewise, the jumbo phage topoisomerases form distinct branches from the cellular versions and show a separation of the DNA-encircling and enzymatic components (Figure 3c). Several jumbo phages also possess the archaeo-eukaryotic version of the peptidyl tRNA hydrolase (PTH2) rather than the version commonly found in their hosts [224]. These observations suggest that the jumbo phages (and the related smaller phages), rather than being degenerations of cellular systems, have evolved from distinct pre-LUCA replicons. Hence, it is possible that they preserve some of the features of these replicons closer to the ancestral form, which were subsequently consolidated in somewhat distinct configurations in the conserved systems of cellular genomes.

Thus, they provide a snapshot of the diverse “experiments” that occurred in the central-dogma-related systems of the early replicons. For instance, it may be inferred that the double-barrel RNAPs were more fluid in the early replicons and comprised of multiple separate subunits, each performing a specific role, that came together to constitute the active RNAP [79,80]. In this regard, the minimal sigma factor and the gp33 TF of group 2 phages provide a possible picture of the ancestral state of the HTH TF that recruited the RNAP to specific promoters. Likewise, the minimal version of the DNA polymerase III module (see below) provides a model of the ancestral versions of this replication enzyme. Thus, versions similar to these phage versions likely duplicated or fused with other domains to give rise to their more complex cellular cognates. Further, the two distinct representatives of the FtsZ/Tubulin family from jumbo phages also suggest that the diversity of functions offered by these self-organizing NTPase proteins (e.g., chromosome segregation and subcellular compartment formation) had already emerged early in evolution and were subsequently recapitulated in the eukaryotic centrosome and primary cilium [225,226].

The minimal version of the cellular DNA polymerase III catalytic module, which we uncovered in this study, maps to more or less just the core catalytic nucleotidyltransferase domain and template DNA strand binding domain (Figure 3c). It is further split into two standalone proteins that are likely to reconstitute complete catalytic enzymes. Notably, the jumbo phages with this version do not contain any of the other domains of DNA polymerase III, instead possess a family B DNA polymerase, like in the archaeo-eukaryotic lineage. Similarly, the C-terminal zinc ribbon of TFIIE, a TF typical of the archaeo-eukaryotic lineage, occurs in jumbo phages that depend on the bacterial RNAP [90,91]. Thus, conserved components of the replication, transcription and translation apparatus that occur separately either in the bacterial or the archaeo-eukaryotic branch of cellular life are juxtaposed with components of the other branch in jumbo phage systems. This suggests that in the pre-LUCA period there was potentially a greater “mixing and matching” of the protein domains performing different central-dogma functions, some of which has survived in the extant phages. It also suggests that the replicons grew by accretion of cooperating genetic elements that provided different core components. Those that acquired different, more complete complements of components leading to self-sufficiency branched off as the two lineages of cellular life. Some of the rest adopted different degrees of parasitic existence and survived as the viral lineages.

This brings us to the final question of what drove the multiple independent increases in genome size resulting in the jumbo phages and whether it informs us in any way about the origins of cellular genomes? A popular proposal is the ratchet model wherein mutations resulting in bigger heads favor growth of genome size through acquisition of new genes. However, the random loss of the genes after they have been fixed would be selected against thereby ratcheting up genome size [139]. While this provides a simple baseline model, which has the advantage of being agnostic to specific genetic features, there are several indications that the underlying landscape of pre-adaptations might play a larger role than implied by this model. The systematic survey of 10,176 complete phage genomes indicates that whereas *Siphoviridae* are present at 2.7 times the frequency of *Myoviridae*, among jumbo phages *Myoviridae* are almost nine times more frequent than *Siphoviridae*. Further, there is no case so far of other relatively common *Caudovirales* groups, like *Podoviridae* or *Autographiviridae*, ever evolving to the jumbo state. This suggests that there is a distinct pre-adaptation among *Myoviridae*, perhaps relating to their DNA injection machinery, that predisposes them to successfully operate at larger genome sizes.

At least 6–7 independent emergences of jumbo phages can be inferred among *Caudovirales* that use the terminase-portal mechanism of DNA packaging [139,227,228]. Strikingly, to date, no prokaryotic virus with lipid membranes internal to capsid, which use the A32/FtsK/HerA-like packaging ATPases, i.e., tecti/cortico-like viruses, are known to have expanded to large sizes [2,229]. In contrast, in eukaryotes, the viruses that accreted on such a tecti-like core gave rise to a wide array of small viruses (e.g., adenoviruses and adomaviruses) and plasmids infecting the mitochondrial descendants of alphaproteobacteria on one hand and the large NCLDVs with genomes reaching sizes comparable to the jumbo phages on the other [2]. Thus, their evolutionary trajectory mirrored *Caudovirales* in terms of occupying the entire spectrum of genome sizes. From among the eukaryotic viruses that accreted around a *Caudovirales*-like core arose the medium-sized herpesviruses that remained at genome sizes comparable to medium-sized precursors of the jumbo phages. These patterns suggest that in addition to the viral pre-adaptations, the host superkingdom has also been a determinant of which viruses expand in genome size.

Several jumbo phages carry several alternatives to countering host defenses and rival phages: for example, they might carry more than one type of RNA ligase and tRNAs or multiple enzymes for NAD^+^ synthesis or potentially alternative enzymes for capsular modification. This parallels the existence of alternative biosynthetic and repair pathways in cellular genomes, suggesting that there has been a certain selection for robustness via functional backups, over and beyond what exists in medium or smaller tailed phages. A parallel might also be struck with cellular genomes wherein within the same clade one might encounter relatively small and large genomes as sister groups—e.g., the gammaproteobacterium *Escherichia coli* has around 4350 genes while the related *Hemophilus influenzae* has only 1800 genes with extensive variation within each genus [230]. A part of this difference arises from the larger genomes coding for a much wider range of metabolic and immune functions. A part of the difference in genome size also arises from the accumulation of selfish genetic elements in the genome. This tendency appears in phage genomes that grow to around the coliphage T4 size [231] and increases as they expand to the size of jumbo phages. These are particularly visible in the form of self-splicing introns (e.g., in the DNA polymerase gene of the *Bacillus* phage G) or inteins (e.g., in the ribonucleotide reductase gene of *Clostridium* phage c-st) in essential phage genes similar to what has been reported for host genomes [232].

Thus, the differences in cellular genome sizes can be seen as occupying a spectrum similar to what in ecology has been described as the r-K selection spectrum—those with smaller genomes are selected by higher replication rates (r), whereas those with larger genomes replicate more slowly but have an entire array of features that make them stronger competitors [132,233]. Similarly, the caudoviral genomes may be seen as being selected along a similar spectrum with fast replication on one end and a stronger capacity to weather host immune attacks and metabolic restrictions on the other. This is borne out by the above observations that jumbo phages are enriched in a diverse array of strategies, both to counter host defenses and withstand physiological limitations arising from environmental conditions. In light of this, the repeated emergence of jumbo phages and their persistence across diverse bacterial groups might reflect an evolutionarily stable strategy in the face of hosts with well-developed immune mechanisms that face a diversity of environmental conditions.

## 4. Conclusions

Using 224 jumbo phages infecting diverse bacteria, including the recently discovered mega phages from *Prevotella*, we provide a comparative genomics overview along with in-depth sequence and structure analysis to provide a synthetic overview of the functionalities encoded by these viruses. Although these phages have evolved from distinct starting points within *Caudovirales*, as reflected in their DNA replication, transcription, and structural systems, they have converged to large genome sizes. This has also been paralleled by similar acquisitions and innovations with respect to metabolic and counter-immunity adaptations, several of which are described here for the first time. We hope that this analysis and the accompanying supporting material provide a framework for future investigations into these viruses.

## Data Availability

The data presented in this study are available in Supplementary Materials.

## Supplementary Materials

The following are available online at https://www.mdpi.com/1999-4915/13/1/63/s1, Supplementary S1.1–1.11: Phyletic vector diagrams for various jumbo phage proteins or protein domain families. Supplementary S2: Multiple Sequence Alignments of various protein domain families described in the text. Supplementary S3: Counts of tRNA genes per genome in representative jumbo phages. Supplementary S4: Relative gene positions of the 100 most frequent protein domains in a set of representative jumbo phage genomes. Supplementary S5: Sequence clusters of all proteins of the jumbo phage genomes analyzed in this study. Also provided is an excel file with complete annotation of all sequence clusters.
